# Nested PCR amplification of the mitochondrial hypervariable region for non-invasive eDNA detection of *Cryptobranchus alleganiensis*

**DOI:** 10.1371/journal.pone.0328633

**Published:** 2025-07-23

**Authors:** Patrick Kurtz, Jorge Santo Domingo, Robert Alexander Pyron, David Wendell

**Affiliations:** 1 Department of Chemical and Environmental Engineering, University of Cincinnati, Cincinnati, Ohio, United States of America; 2 United States Environmental Protection Agency, Cincinnati, Ohio, United States of America; 3 Department of Biological Sciences, The George Washington University, Washington, DC, United States of America; Niigata University of Pharmacy and Medical and Life Sciences, JAPAN

## Abstract

Environmental DNA (eDNA) has proven an invaluable tool in detecting elusive, endangered, or otherwise hard-to-identify species in aquatic ecosystems. The Eastern hellbender salamander (*Cryptobranchus alleganiensis*) is a fully aquatic amphibian with an increasingly threatened population due to its reliance on exceptional surface water with high levels of dissolved oxygen and low sediment. *C. alleganiensis* habitat and animal loss has been documented through conservation work, resulting in the U.S. Fish and Wildlife Service recently recommending *C. alleganiensis* as endangered under the Endangered Species Act. Recent natural disasters in population strongholds have emphasized the need for continued conservation efforts, including reliable, non-invasive methods for monitoring *C. alleganiensis* populations. Using eDNA extractions from environmental waters, we have developed a nested polymerase chain reaction (PCR) detection strategy targeting the control region of the *C. alleganiensis* mitochondrial genome. We demonstrate the efficacy of our nested primer set for the detection of *Cryptobranchus* salamanders in the Ohio River watershed with conventional PCR, quantitative PCR, and digital droplet PCR methods, and rigorously challenge these primers against diverse aquatic amphibian DNA templates found within their habitat using DNA sequencing to confirm the specificity of our target amplicons. Our nested PCR approach overcomes several shortcomings of previous eDNA detection methods for *C.* alleganiensis, most notably an order of magnitude improvement in the limit of detection for animal DNA in the field.

## 1. Introduction

Amphibian species have undergone precipitous population decline in recent years due to a myriad of compounding factors including fungal pathogens, large-scale agricultural practices, and habitat loss from changing global and environmental conditions [1–4]. For example, Hurricane Helene’s devastation of Western North Carolina impacted century-old strongholds for *C. alleganiensis* populations—with several reports documenting deceased hellbenders in the aftermath of the flash floods [5]. Acute devastation of habitats like these remains something that conservationists and scientists must prepare for—as climate change will continue to deliver larger, more intense storms which destabilize essential conservation areas. The chronic effects of higher water temperatures from climate change, compounded with sedimentation from farming activity has promoted pathogenic infection in amphibians in general [6,7] and impaired breathing specifically, as *C. alleganiensis* relies on cutaneous gas exchange for respiration [8] making them particularly vulnerable to changes in water quality [9,10].

Environmental DNA (eDNA), generally defined as cellular DNA shed by living organisms in the environment [11], has proven to be an effective tool to detect organisms that are difficult to locate with conventional field surveys. Methods based on the detection of specific gene targets in eDNA extracts (e.g., Polymerase Chain Reaction (PCR) and DNA Sequencing-based methods) are useful for detecting rare and reclusive aquatic species because of their low-cost, exquisite sensitivity, specificity, and performance [12–19]. PCR is a powerful method for nucleic acid amplification with several variations including quantitative PCR (qPCR) and digital droplet PCR (ddPCR), which each allow for the quantification of the target DNA present in a sample. eDNA methods have been established for a variety of natural habitats including freshwater streams, lake sediments, as well as terrestrial habitats like tree bark, topsoil, and even spider webs [20–23]. Since accurate detection of aquatic taxa is vital to their conservation, the design of highly specific, thoroughly tested primers is vital to eliminate false positive data (i.e., amplification of non-target sequences from diverse sources of eDNA present) and overcome inhibition posed by varied water quality contents. The various challenges posed by eDNA detection methods require strict protocols in every facet of sample collection, extraction, amplification and evaluation to provide accurate detection of the target animal’s DNA [24,25]. Both nuclear and mitochondrial DNA (mtDNA) may be targeted in eDNA detection, but the markedly higher copy number of mitochondrial genomes per cell makes mtDNA genes more attractive targets [26,27].

At a fraction of the size of genomic DNA and circular in structure, mtDNA is thought to persist for longer periods of time in the environment due to the added protection of the organelle membranes [28,29]. The genes within the mitochondrial genome are essential to cellular respiration in eukaryotes and thus, are conserved in genetic structure, typically encoding for two rRNAs, 22 tRNAs and 13 polypeptides. However, the order of the genes, codon usage, and non-coding regions all serve as sources of differentiation between species and potentially within the same species depending on the mtDNA genetic diversity [28]. Of these mitochondrial regions, the D-loop is one of the most valuable in providing discerning sequence information. Functioning as a control region for replication, this genetic site often contains repeated, variable sequence elements as well as conserved areas that mark the start/stop of the DNA replication process. Because the D-loop is a non-coding region (commonly referred to as the Hyper Variable Region or HVR), it accumulates mutations at a higher rate than other regions of the mitochondrial genome [28], and consequently the HVR has demonstrated utility in forensic and ancient DNA analysis—uniquely identifying shared maternal inheritance [30], differentiating evolutionary haplotypes [31,32], and detecting rare aquatic species from mtDNA found in the environment [33].

Targeted eDNA primer design often focuses on the mtDNA cytochrome B (CytB) gene to differentiate between species because of its sequence specificity and the abundance of available reference sequences for comparison [12,19]. However, depending on the species in question, the CytB gene does not always provide the most information for differentiation when compared to other molecular targets within mitochondrial genomes [34,35]. Multiple eDNA-based methods to detect *C. alleganiensis* have been reported [12,36,37], with the qPCR method developed by Spear [12] serving as the most widely used [38–41] and as a result, the method we chose for our comparison.

Nested PCR, a variation of conventional PCR [42], adds additional specificity to a PCR reaction as it allows coupling of broad amplification of related taxa with specific detection of the taxa of interest. Recently, nested PCR has been used in eDNA detection [32,43–46] but remains under-utilized compared to other methods [12–19]. While qPCR and more recently, ddPCR, remain the gold standard for DNA quantification, these methods can require more expensive reagents such as TaqMan probes, and specialized thermal cyclers when compared to conventional PCR—which relies on gel electrophoresis and in some cases DNA sequencing for confirmation of correct eDNA identification [24].

**Fig 1:**
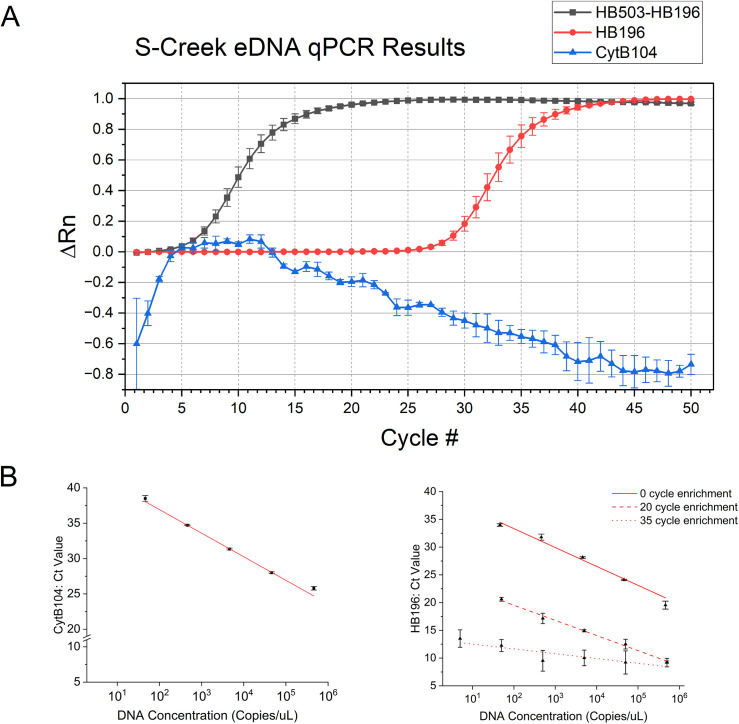
S-Creek eDNA qPCR results. (A) qPCR results using the first S-Creek DNA extraction (0 ft) as template with the CytB104 primers/probe, the HB196 primers without the first step enrichment, and the HB196 primers using HB503-enriched PCR product as template (B) qPCR standard curves for CytB104, HB196, HB196 with 20 cycles of HB503 template enrichment, HB196 with 35 cycles of HB503 template enrichment. Template concentrations range from 500,000 copies/µL to 5 copies/ µL (Error bars represent standard error, n = 3).

In this study, we sought to investigate current methods of *C. alleganiensis* eDNA detection, and improve upon them, developing a novel molecular method that we demonstrate to be sensitive, robust in the field, and without the vagaries of off-target amplification from current primer sets, making it more reliable. Recently, the most cited *C. alleganiensis* qPCR method developed by [12], referred to hereon as the “CytB104” primers for their 104 bp CytB target, were applied in a *C. alleganiensis* conservation study conducted in West Virginia where distilled water controls reportedly produced false positive detection results [41]. In our application of the CytB104 primers, we also observed off-target amplification in both field and pure animal DNA extractions (see Results Figs 1D, 3) when employed as previously described [12]. To overcome current limitations with CytB104, we developed a nested PCR method targeting the D-loop control region of the hellbender’s mtDNA. This new primer set offers an additional molecular target, but because of the HVRs, also provides greater opportunity for intraspecific genetic differentiation [47,48].

**Fig 2:**
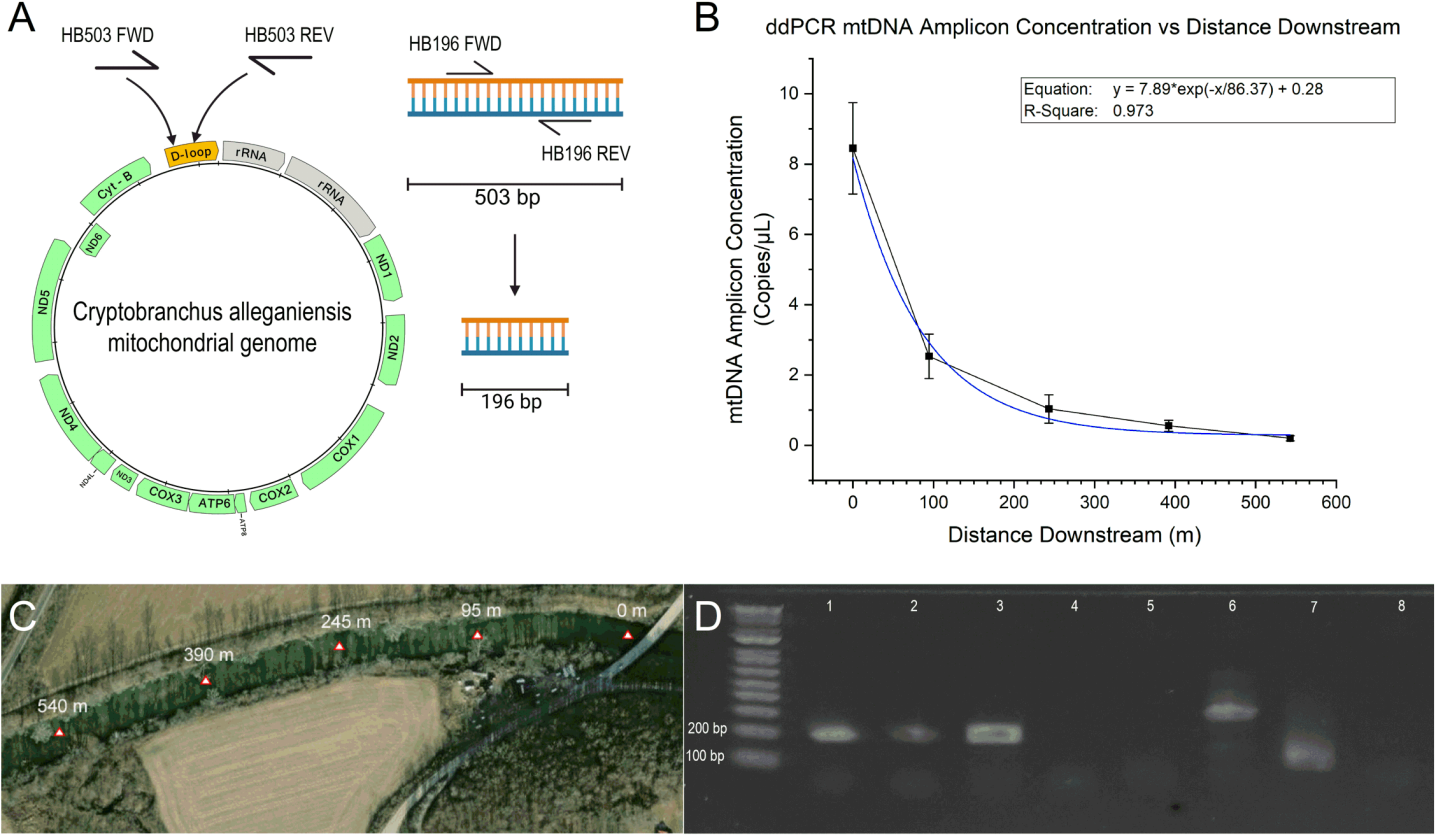
ddPCR mtDNA amplicon concentration as a function of distance. (A) DNA schematic of the HB primer sets [67] (B) ddPCR quantification results for each sample collected along S-Creek in November of 2017* (Blue trendline: exponential decay equation fit using Levenberg–Marquardt algorithm) (C) Satellite view of S-Creek and each sampling location** Credit: U.S. Geological Survey (D) PCR product from HB196 primers on HB503 enriched S-Creek eDNA extractions (Lanes 1: 0 m, Lane 2: 245 m, Lane 3: Positive control, Lane 4: NTC) and CytB104 primer on S-Creek eDNA extractions (Lanes 5: 0 m, Lane 6: 245 m, Lane 7:Positive control, Lane 8: NTC). * Error bars represent standard error, n = 5. **The weeks following the sampling event at S-Creek in 2017 brought heavy rains and flooding and the detected hellbender populations were confirmed to no longer reside there.

**Fig 3:**
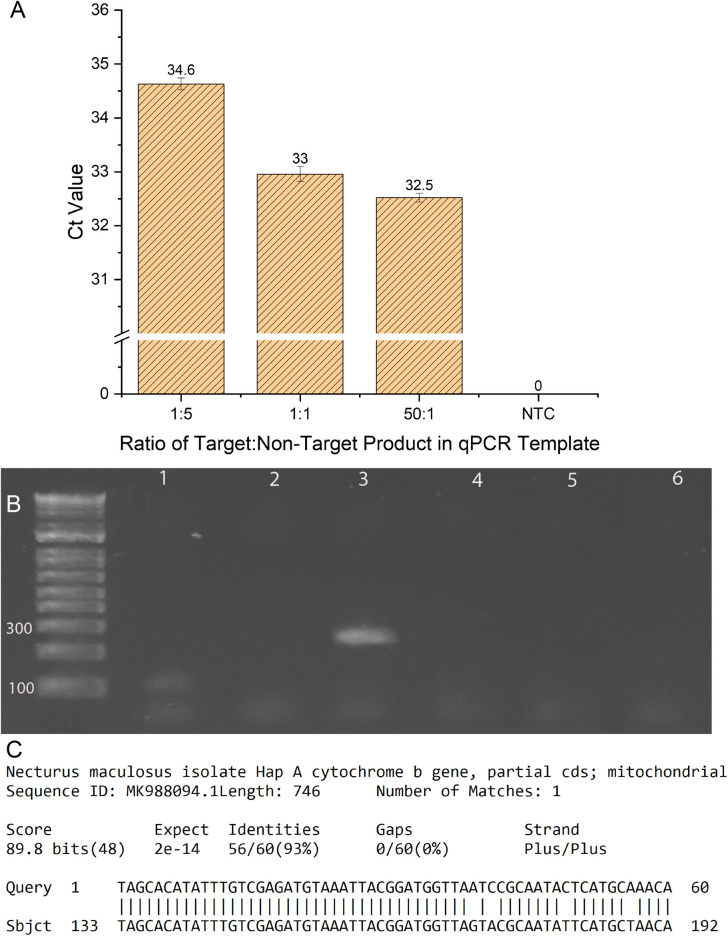
PCR effeciency for CytB104 primers in the presence of self-generated off-target amplicon. (A) Ct-value results from qPCRs conducted with three different ratios of Target: Non-Target DNA* (B) PCR results using the CytB104 primer set on 4 *N. maculosus* DNA templates (Lanes 1-4, No Template Controls Lanes 5-6). ~ 250 bp band in lane 3 was gel-purified and used as off-target template for qPCR analysis displayed in A (C) NCBI BLASTn results for the ~ 100 bp product gel-purified and Sanger sequenced from CytB104 PCR (Fig 3B, Lane 1) indicating exact homology with the *N. maculosus* cytochrome-B gene. *Error bars represent standard error, n = 3.

Our nested PCR method consists of outer primers (HB503) designed for relatively conserved regions of the D-loop, and a highly hellbender-specific inner primer set (HB196). As previously established [43,44], nested PCR delivers increased sensitivity from PCR’s enrichment when compared to a single primer sets performance on eDNA samples of unknown inhibitory content. We validate the performance of our new primers starting with DNA extractions from captive bred hellbenders (Columbus Zoo, OH), followed by eDNA extractions from streams within the Midwest and Central Ohio River Valley. Next, we challenged these primers against DNA extractions from the common mudpuppy *(Necturus maculosus*) to examine possible detection interference from an abundant, co-localized amphibian. Indeed, *N. maculosus* is the only other fully aquatic salamander found in much of the Eastern hellbender’s geographic range [49,50], and while the evolutionary divergence between the two species is significant, there does remain some mtDNA homology (Table 1) that should be considered to avoid off-target amplification. To extend this potential off-target analysis, we also included samples from a second salamander found in the Ohio River watershed, the Northern dusky salamander (*Desmognathus fuscus*). *D. fuscus* is semi-aquatic, potentially occurring in the same streams as the hellbender, but without any likely hybridization. Additionally, *Desmognathus* species have a complex history of hybridization and genomic recombination between species within the genus [51,52], with the potential for an evolving palette of off-target nuclear and mitochondrial sequences.

**Table 1:** Primer sequence comparison.

Primer Description	Primer Sequence	% Identity
*C. alleganiensis* HB503 FWD Primer:	5’-3’: ACGCATAACTGAGTCTGCCC	
*N. maculosus* mtDNA Sequence^*^:	5’-3’: CAGCATAACTGCACTAAATT	45
*D. fuscus* mtDNA Sequence:	5’-3’: CCCCATAACTG-GTTTGATG	53
*C. alleganiensis* HB503 REV Primer:	5’-3’: AGCATTTTCAGTGCTGTGCT	
*N. maculosus* mtDNA Sequence^*^:	5’-3’: GGCATTTTCACTGCCACACT	70
*D. fuscus* mtDNA Sequence:	5’-3’: GGCATTTTCAGTGCCATATT	75
*C. alleganiensis* HB196 FWD Primer:	5’-3’: CTGTGTGGTCAACCA-ACATAAA	
*N. maculosus* mtDNA Sequence^*^:	5’-3’: TTGTGATCTCAGTTCGACATCGT	39
*D. fuscus* mtDNA Sequence:	5’-3’: CTGTGAGGTCAACAG-ACATATA	50
*C. alleganiensis* HB196 REV Primer:	5’-3’: CTTTTCTTGATAATTCAGTTCCGG	
*N. maculosus* mtDNA Sequence^*^:	5’-3’: TTACTCTTAATAAATCATTTTTGT	63
*D. fuscus* mtDNA Sequence:	5’-3’: AATTATT-GGGAAAATGGTTTCGT	52
*C. alleganiensis* CytB104 FWD Primer:	5’-3’: GTTTGCATGAGTATTRCGGATT	
*N. maculosus* mtDNA Sequence^*^:	5’-3’: GTTAGCATGAATATTGCGTACT	77
*D. fuscus* mtDNA Sequence:	5’-3’: GTTTGCGTGAATATTCCGTATA	77
*C. alleganiensis* CytB104 REV Primer:	5’-3’: TCGCTATRCATTATACAGCAGATACA	
*N. maculosus* mtDNA Sequence^*^:	5’-3’: TGGCTATACATTATACAGCAGACACA	88
*D. fuscus* mtDNA Sequence:	5’-3’: TAGCTATACATTATACAGCAGATACC	88
*C. alleganiensis* CytB104 Probe:	5’-3’: CATCTCGGCAGATATG	
*N. maculosus* mtDNA Sequence^*^:	5’-3’: CATCTCGACAAATATG	88
*D. fuscus* mtDNA Sequence:	5’-3’: CATCACGGCAAATGTG	81

*C. alleganiensis* Primer Comparison with mtDNA sequences of Appalachian salamanders: Common Mudpuppy (*N. maculosus*), Northern Dusky Salamander (*D. fuscus*).

Degenerate Nucleotide *N. maculosus* Nucleotide Miss *D. fuscus* Nucleotide Miss.

* Accession #: PV068071, PV068072.

## 2. Methods

### 2.1. Study design

Environmental sampling in this study followed the guidelines from eDNA studies available at the time of the research start date [17,24,26]. Additionally, previous *C. alleganiensis* eDNA studies [12,37–39] were evaluated for applicable sampling methods and laboratory analysis. Field collection of stream samples proceeded in accordance with standard practices from these studies [12,17,24] wherever possible as 1 L samples were taken from the middle of streams and filtered to obtain maximum DNA per volume (see Methods 2.3 for detailed sampling methods). Preliminary PCR surveys using direct animal DNA and eDNA from 2 creeks in south central Ohio (12 sample locations) revealed a need for an improved PCR detection method for *C. alleganiensis*, (see Methods 2.4 for detailed PCR methodology) and Ohio surface waters were used to test and refine said method.

S-Creek, a creek in south-central Ohio, was identified as an ideal setting to establish the eDNA detection limit by distance for the HB503/HB196 primers assay due to the known release of a captive-bred animal and engineered concrete burrow near this location. The historically limited distribution of *C. alleganiensis* in this stream before this study supported the experimental unlikelihood of additional Eastern hellbenders residing downstream of the confirmed animal’s location. eDNA samples collected at S-Creek and other Ohio/Indiana streams, as well as positive/negative control DNA extractions (see Methods 2.3), were extensively employed in conventional PCR, qPCR, and ddPCR to validate the use of the HB503/HB196 nested PCR method for *C. alleganiensis* by comparing its performance to the published CytB104 qPCR method. Limits of detection, off-target amplification events, and sequence specificity were then examined for each primer set using the methods that follow.

### 2.2. Primer design

Primer design began with NCBI BLAST analysis in conjunction with a published, partial *C. alleganiensis* mitochondrial genome [53] (GenBank: GQ368662.1). We searched for conserved regions outside of the larger D-loop target and inner primers specific to our target species. The D-loop control region was selected specifically for its hyper variable region, which provides a wealth of sequence variation between and even within species [47]; while the mtDNA genetic diversity for the Eastern hellbender remains unknown, the control region has the potential to be used as a differentiator between hellbender populations like it has for other species [54–56].

First, we developed two primer sets targeting conserved portions of the D-loop we labeled “HB609” and “HB503,” referring to the amplicon length of the initial targets. The HB609 assay produced little amplification when compared to that of HB503 on direct animal extractions, suggesting lower sensitivity, and therefore was not used in further studies. The HB503 primers flank the control region, binding to relatively conserved areas of the mitochondrial genome, allowing the HB503 PCR to serve as a general enrichment step for aquatic mitochondrial DNA—similar to other methods of DNA enrichment [57–59]. The inside nested primers (HB196) were designed in silico to bind to consensus sequences within the more divergent HVR region of *C. alleganiensis* providing specificity against off-target amplification of closely related taxa. BLASTn was employed extensively throughout this process to first screen potential sequences applicable to *C. alleganiensis* which are not homologous to any known salamander in its habitat range (see Table 1 for primers, including off-target analysis, and S2–S5 Files for BLASTn results for each primer). *C. alleganiensis* shares significant mtDNA homology with several giant Asian salamanders of the *Andrias* genus—which reside exclusively in Asia—so this genus was excluded from the BLASTn results of the HB196 primers to show results more relevant to North American freshwater ecosystems. Multiple pairs of primers were designed and screened against positive animal controls throughout this process, and the HB503/HB196 primers provided the most robust and sensitive detection among the designs considered.

### 2.3. Sample collection and DNA extraction

To obtain *C. alleganiensis* eDNA in the field, stream grab samples were collected in sterile, 1 L Nalgene bottles with new, disposable gloves (each sample). Samples were taken from the middle of the stream at a depth of 1–3 feet below the surface, depending on the depth of the stream. Multiple 1 L samples were collected at each site/distance in accordance with previous studies [12,17,24,26,40], resulting in at least 2 field replicates per sampling site with duplicate eDNA extractions per replicate if less than 1L passed through the filter before clogging due to biomass and other solids. Distances between samples were measured using a Garmin GPSMAP® 64s. Field blanks using lab-generated NanoPure water were opened at each site and processed through the same filtration method as the environmentally collected water samples. All samples and field blanks were transported in ice coolers to the lab within hours of collection and filtered in the lab through a 0.45 um, 47 mm diameter nitrocellulose membrane secured in sterilized stainless-steel holders and later, through disposable plastic filtration holders. Each filter (eDNA filters and field blanks) was transferred to an individual sterile 6 cm petri dish and stored at −20°C until eDNA extraction. eDNA collection and handling protocols followed best practices as previously established [24,26], with samples typically filtered and extracted the same day.

Direct *C. alleganiensis* tissue swabs were collected with the help of the Columbus Zoo staff by means of nitrocellulose filter swabs applied directly to the skin of the animals and DNA extractions performed as described previously for the environmental filter samples. Additional positive controls were obtained by grab sampling water from the tanks of captive bred hellbenders, but direct swabs yielded more animal mtDNA.

Qiagen Power Water Kits (Germany) were used to extract the genetic material collected on the filter. Filters were first cut into strips using a new sterile razor for each sample prior to addition into the kit’s paper/cell shredding silica beads. The DNA extraction was performed as defined in the kit protocol and eluted in 100 uL volumes of EB buffer. Finally, the concentration of nucleic acids was measured using a Thermo Scientific NanoDrop with yields typically in the range of 0.5–10 ng/µL. The eDNA eluate was kept at 4°C until further analysis with PCR.

### 2.4. PCR methods: HB503, HB196 primers, CytB104 primers and probe

#### 2.4.1. Nested PCR: HB503/HB196 primers.

The nested PCR method consists of an outer primer set (HB503 Forward/Reverse) that targets a 503 bp region within the control region of the *C. alleganiensis* genome. The inner primer set (HB196 Forward/Reverse) targets a 196 bp region within the 503 bp of the first round PCR. Both of these amplicon sizes are larger than average eDNA target designs [12–19], including the predicted, ideal amplicon size for greatest qPCR efficiency [24,60]; however, the cost of any decreased efficiency was viewed as offset by the benefits of greater sequence information as a potential animal identifier. All primers were synthesized by Eurofins Genomics, and TaqMan probes were obtained from Applied Biosystems. The HB503 and HB196 primer sequences are as listed:

HB503 Forward: 5’ ACGCATAACTGAGTCTGCCC 3’
HB503 Reverse: 5’ AGCATTTTCAGTGCTGTGCT 3’
HB196 Forward: 5’ CTGTGTGGTCAACCAACATAAA 3’
HB196 Reverse: 5’ CTTTTCTTGATAATTCAGTTCCGG 3’

The conventional PCR protocol for the HB503 primer set was first optimized using New England Biolabs 1TAQ DNA Polymerase. The optimized protocol consisted of an initial denaturation at 94°C for 30 seconds, followed by 35 cycles of denaturing for 20 seconds at 94°C, annealing for 30 seconds at 62°C, and an extension step for 40 seconds at 68°C. A final extension was held for 5 minutes at 68°C and the reaction was held at 4°C until analyzed with gel electrophoresis. The HB196 reaction’s optimized conditions were developed using New England Biolabs Q5 HiFi Polymerase and consisted of an initial denaturation for 30 seconds at 98°C, and 30 cycles with a 10 second denaturation at 98°C, a 12 second anneal at 60°C, and a 5 second extension at 72°C followed by a final extension for 2 minutes at 72°C. Each reaction optimization used 1 µL of 10 uM primer stocks per 25 µL reaction and 1 µL of template DNA with a concentration range of 1–5 ng. PCR products were purified using Qiagen’s MinElute or QiaQuick Gel Extraction kits as needed. Conventional HB503/HB196 PCR reactions on eDNA templates were run in triplicate.

#### 2.4.2. Quantitative PCR.

We employed the CytB104 qPCR method [12] exactly as described, using 50-rounds of qPCR on an Applied Biosystems 7500 with a 15-minute hot start at 95°C followed by cycling conditions of 94°C for 1 minute and 60°C for 1 minute using Qiagen’s QuantiTect Multiplex master mix in conjunction with a TaqMan probe (see Table 1 for sequences). Our HB196 primers were also employed in qPCR using the exact same cycling conditions and qPCR master mix kit, but with SYBR I used for quantification instead of a TaqMan probe. SYBR based qPCR amplicons were verified via melt curves, gel electrophoresis, and Sanger sequencing. qPCR was employed on eDNA templates from S-Creek using both the CytB104 and HB503/HB196 primer sets. As the CytB104 method relies on the specificity of the TaqMan probe for *C. alleganiensis* detection, qPCR was used as a direct comparison between the two primer sets—with the HB196 primer set applied directly with eDNA templates, and with enriched template from an HB503 PCR. Each qPCR reaction was completed with replicates ranging from n = 3–6, depending on eDNA template concentration.

#### 2.4.3. Digital droplet PCR using HB503/HB196 primers.

Digital droplet PCR was employed with the HB503/HB196 primer sets to quantify eDNA in several samples collected in a Central Ohio Creek (we reference as S-Creek to protect animal locations) shortly after the hellbender’s mating season in October of 2017. The HB503 primers were used on each respective S-Creek eDNA extraction in 20-cycle PCRs to enrich the *C. alleganiensis* mtDNA present in the extractions. 20 µL reactions were performed using 1 µL of template DNA (purified HB503 PCR product), 1.1 µL of each HB196 primer’s 10 µM stock solution, 11 µL of Bio-Rad Qx200 Evagreen Supermix, and 7.8 µL of nuclease-free water. The samples were transferred to the Bio-Rad DG8 cartridge where oil was added and then emulsified in the Bio-Rad QX200 Droplet Generator. The emulsified samples were then transferred to a 96-well plate and loaded into the Bio-Rad C100 Thermocycler. The PCR conditions consisted of a 5-minute initial denaturation at 95°C followed by 40 cycles consisting of a 30 second denaturation at 95°C and a 55°C anneal for 1 minute. The samples were then brought to 4°C for 5 minutes, 90°C for 5 minutes, and held at 12°C thereafter. While ddPCR does not require an explicit standard curve, at least 10,000 droplets from each emulsified PCR reaction are required to produce statistically significant results under a Poisson distribution and be included in the subsequent data analysis [46,61,62]. Therefore, only reactions that met this requirement were included in our analysis with typical droplet yields around 14,000–20,000 (see Fig S26 in S1 File).

### 2.5. qPCR standard curve and limit of detection

PCR products from the CytB104 and HB196 reactions on positive control (PC) *C. alleganiensis* swab extractions were purified (Qiagen QiaQuick PCR Clean Up) and Sanger sequenced to verify the PC amplicon, then concentrated to 10–15 ng/ µL with Qiagen MinElute. Similarly, pure template for the HB503-enriched reactions was generated from mtDNA sequences containing the entire control region and CytB genes of *C. alleganiensis*. The purified product from each of the CytB104, HB196, and HB503 PCRs was then used as template to establish limits of detection for each method using dilutions spanning 10 orders of magnitude from 5 x 10^10^ copies/µL to 5 copies/µL. Dilutions were completed in nuclease-free water (Invitrogen), thus representing a best-case scenario with pure dilutions of respective target DNA sequences. HB503 PCRs were conducted with the same temperature/time conditions as described in Methods 2.4 with 20 cycle and 35 cycle enrichment reactions on each respective template dilution. qPCR then proceeded as described previously [12] for each respective primer set, using the Applied Biosystems 7500, Qiagen Quantitect Multiplex PCR mix with a hot-start, 50 cycle, two-step reaction. Each purified product dilution reaction had a final volume of 15 µL and was completed in triplicate.

### 2.6. Off-target amplification

The HB503/HB196 primers and the CytB104 primers were tested against various sources of negative control DNA, including eDNA extractions from streams with no *C. alleganiensis* (combined sewer overflow concrete raceway, Duck Creek, Cincinnati OH [63]) and DNA extractions from other Ohio salamanders (*N. maculosus*, *D. fuscus*). Positive control (PC) sources of DNA included extractions from swabs applied to captive *C. alleganiensis*, and tank water of these animals.

We employed BLAST for primer design and molecular target verification in conjunction with Sanger sequencing of PCs and eDNA-derived nested PCR amplicons. In this way we verify that the product generated in a qPCR, ddPCR, or nested-PCR is from *only* the target animal, as Sanger requires a single, pure DNA product to generate unambiguous chromatograms. Similarly, off-target amplicons and amplicons generated from negative control templates using CytB104 primers were gel-purified, analyzed with Sanger sequencing and NCBI BLAST to elucidate their origin. Some of these off-target sequences were identified directly through nucleotide BLAST, but for others, no direct nucleotide match was found even under highly dissimilar allowances. In those cases, BLASTx and ORFfinder were used in conjunction with BLASTp to analyze any possible coding regions within the sequence to glean potential protein sequences from a species of origin.

Lastly, because off-target amplification was observed using the CytB104 primer set in conventional PCR, an experiment was devised to analyze the possibility of signal interference in qPCR from these off-target products. This was done because the CytB104 qPCR method benefits from the specificity provided by the TaqMan probe. While there may be an assumption that off-target products do not impact a qPCR reaction when using a TaqMan probe, diversion of nucleotide resources to off-target products during the PCR reaction may occur. This could lead to a potential loss in sensitivity of target detection in the theoretical presence of both target and non-target eDNA [64]. To examine the results of such a diversion of reaction resources, an off-target amplicon generated with the CytB104 primer set from a *N. maculosus* DNA sample was purified and spiked into qPCR reactions, where target DNA (sequence-verified CytB104 PCR product) was combined with off-target DNA at three different ratios of Target to Non-target DNA by copy number (1:5, 1:1, 50:1). These template mixtures were selected based on previous off-target investigations [64] and were designed to test potential scenarios where cohabitating animals at different distances and/or population numbers are present alongside *C. alleganiensis* in the stream with the potential to impact subsequent eDNA assays.

### 2.7. *N. maculosus* control region PCR and sequencing

To obtain sequence information on the control region of *N. maculosus*, we sequenced a 2500 bp PCR fragment by primer walking and Sanger sequencing (see Method S2 in S1 File) covering the control region for this species using animal samples (genomic DNA extractions from live animal tail clippings) collected within the Great Lakes and Ohio River Valley (generously donated by Dr. Greenwald). The sequences were uploaded to NCBI under accession numbers PV068071, PV068072.

### 2.8. Ethics statement

Swabs from the protected species *C. alleganiensis* were collected by trained personnel at the Columbus Zoo under their Ohio Center for Wildlife Conservation. *D. fuscus* samples were collected under George Washington University IACUC: A2022-004. DNA extractions from live specimens of *N. maculosus* from Dr. Greenwald were approved by the Ohio Department of Natural Resources Division of Wildlife and collected in accordance with the Animal Ethics Committee of the Cleveland Museum of Natural History. No permits were required for field site access for this study as all stream access was on public land.

## 3. Results

### 3.1. Primer analysis and method validation on eDNA extractions

We first sought to establish the limit of detection (LOD) for both the CytB104 primers/probe, the HB196 primers without HB503 enrichment, and the HB503/HB196 nested workflow with varying degrees of template enrichment in a qPCR context. The LOD in qPCR is defined as the lowest standard of dilution that is amplified in over 95% of replicates [25]. The experimental LOD for both the CytB104 primer set and the HB196 primer set was 50 copies/µL (Fig 1B)—where every replicate amplified in qPCR. Even without the use of the HB503 PCR enrichment step, the HB196 primer set was more sensitive than CytB104, producing slightly lower Ct-values per copy of template DNA, with a Ct-value of 34 for the 50 copies/µL concentration (HB196), compared to a Ct-value of 38.5 for the same template concentration (CytB104) (Fig 1B). Importantly, the HB196 primers in this comparison were used directly on the diluted eDNA templates, without the HB503 enrichment so as to confirm their utility as a stand-alone primer set.

The application of HB196 in conjunction with an HB503 enrichment demonstrates a considerably more sensitive presence/absence test than either HB196 or CytB104 alone. Notably, the 35 round HB503 enrichment produced a limit of detection an order of magnitude more sensitive at 5 copies/µL than that of either single primer set’s performance—demonstrating the nested method’s superior low-copy detection on eDNA samples (Fig 1A) and purified template DNA (Fig 1B). Fig 1B shows that the enrichment step can introduce additional uncertainty in absolute quantification, observed in the flattening Ct standard curve as the enrichment cycles create saturating amounts of HB196 HVR template DNA (Fig 1B)—a result consistent with past studies examining preamplification’s effect on DNA detection [65,66].

Fortuitously, we identified *C. alleganiensis* in S-Creek in an early sampling event during the end of the animals mating season in 2017. The water conditions were low flow and clear, and thus, ideal for a limit of detection analysis with ddPCR. Using samples collected immediately downstream of a manufactured hellbender-burrow and then sequentially at approximately 310 feet (95 m), 800 feet (245 m), 1280 feet (390 m), and 1780 feet (540 m) downstream, we used ddPCR in conjunction with the HB503/HB196 primer sets to detect the presence of *C. alleganiensis* eDNA. The ddPCR results demonstrated animal detection at approximately 800 ft (245 m) downstream of a live animal burrow (Fig 2B, 2C), with results indicating a concentration around 0.5 and 0.1 copies/µL for the 390 m and 540 m samples, respectively—equating to positive detection observed in only 2–4 droplets out of the 16-17k for the most distant sample. ddPCR was specifically employed in this circumstance because of its superior low copy number sensitivity, and this concentration of positive droplets approaches the threshold of ddPCR’s detection capabilities within an eDNA context [57].

To further compare the two primer sets, both were employed in conventional PCR and qPCR reactions using the S-Creek eDNA extractions as template and employing the previously referenced methods. Surprisingly, the CytB104 primer set failed to detect any *C. alleganiensis* mtDNA in the S-Creek extractions but did produce off-target amplification as seen in the faint bands of 200–300 bp (Fig 2D, Lanes 5–6), which may have contributed to this lack of target amplicon generation. The HB503/HB196 nested PCR method produced a clear band around 200 bp on the eDNA extract collected furthest upstream and closest to the animals, and a faint band of the same length from the third sample (800 ft (245 m) as examples)—indicating successful detection of *C. alleganiensis* present (Fig 2D, Lanes 1–2), consistent with ddPCR results and the dilution/degradation/loss of the animal eDNA downstream.

Applied Biosystems 7500 qPCRs were run on the same S-Creek extraction templates for additional comparison (Fig 1A). The CytB104 primer/probe set was used as described previously. The HB196 primers were used both directly on S-Creek eDNA (without an enrichment PCR reaction), and on enriched DNA derived from HB503 PCRs (35 cycles) of the same S-Creek eDNA samples for comparison. In confirmation of the previous presence/absence conventional PCR that generated a non-cytochrome B fragment (Fig 2D lane 5–6), the CytB104 method failed to generate a qPCR Ct above background, while the HB196 primers’ reactions resulted in a Ct value of approximately 30 cycles, (produced without any HB503 PCR enrichment), and a Ct value of around 8–9 cycles for the HB196 primers with enriched HB503 PCR DNA as template (Fig 1A). The HB503 primer set, therefore, improved the efficiency of the HB196 qPCR reaction on eDNA template, decreasing the Ct value by approximately 22 cycles when the HB503 enrichment PCR reaction was included.

### 3.2. Off-target amplification analysis

In a similar manner to eDNA samples, the HB503 primers amplified mtDNA fragments with a wide range of lengths from the *N. maculosus* DNA samples, representing D-loop fragments of various sizes (see Fig S6 in S1 File). The subsequent nested PCR—using purified DNA from the HB503 PCR as template for the HB196 primers—produced no amplification either at the target size around 200 bp, or of any other molecular weight (see Fig S7 in S1 File), thus, supporting the primers’ specificity. In contrast, the CytB104 primer set produced off-target amplicons on a variety of *N.* maculosus DNA templates (Fig 3B, Figs S9 and S10 in S1 File). Sanger sequencing and BLAST results from one of these amplicons (Fig 3C) confirm that the cytochrome-B DNA sequence from *N. maculosus* was amplified with the CytB104 primer set using the published PCR protocol [12]. The CytB104 primers also generated an amplicon of approximately 250 bp on a single *N. maculosus* animal DNA extraction, which we estimated to be too large for the CytB target (Fig 3B, Lane 3). The nucleotide sequence from this amplicon, elucidated through gel-purification and Sanger sequencing, did not produce any definitive results from BLASTn indicating it may not be *N. maculosus* in origin. For further analysis, we used NCBI’s ORFfinder and searched under the resulting protein sequences through BLASTp. Among the lowest E-values was a protein sequence for a Nitric Oxide Reductase from the S*tutzerimonas* genus (see Figs S20–S22 in S1 File). Because this result implicates a protein involved in cellular respiration related to CytB, we anticipate the CytB104 primers are capable of amplifying bacterial genes as well. However, it is unlikely (given the sequence) that this off-target amplicon would react with the TaqMan probe, but it could decrease or impair detection of *C. alleganiensis* targets by way of PCR interference. Additionally, all primer sets were tested on *D. fuscus* tissue DNA extractions (Accession: GSU-27996), and no off-target amplification was observed (see Figs S14–S16 in S1 File).

After examining off-target amplicons from the CytB104 primers, we next tested if the off-target product, specifically the ~ 250 bp amplicon (Fig 3B, Lane 3), would impact the detection of the intended molecular target using purified target CytB mtDNA from hellbender at varying concentrations (Fig 3A). Ratios for Target: Non-Target template DNA (by copy number) of 1:5, 1:1, and 50:1 were implemented based on a previous work related to relevant concentrations of off-target inhibition in qPCR [64]. Unsurprisingly, the presence of off-target template significantly interfered with the efficiency of the qPCR reaction, producing an increase in the necessary cycle number relative to controls with lower concentrations of the interfering sequence (Fig 3A).

To further investigate potential off-target interactions within our mtDNA target region, we generated amplicons spanning the control region of the *N. maculosus* mitochondrial genome (from 12s-rRNA gene to the CytB gene, accession numbers: PV068071, PV068072, Method S2 in S1 File) for 2 animals using DNA extractions generously provided by Dr. Greenwald (see Fig S13 in S1 File). After this, we compared the hypervariable regions between *C. alleganiensis* and *N. maculosus* to provide insight into the HB503 primer sets’ specificity and assess the potential of any future off-target amplification on *N. maculosus* DNA templates based on sequence homology. The ClustalΩ algorithm [68], a multiple sequence alignment program, was used to analyze sequence relatedness between the *C. alleganiensis*-targeted primer sequences examined in this study and their equivalent D-loop and Cyt-B mtDNA sequences from *D. fuscus* and *N.* maculosus. Table 1 displays the results of this analysis demonstrating the specificity of the HB503/HB196 primer sets with significantly lower shared sequence identity to off-target animals.

## 4. Discussion

### 4.1. eDNA quantification considerations and limits of detection

The HB503/HB196 primer sets rely on the high specificity of the nested primers to produce detection without the need for a fluorescent probe, allowing for an easily scalable reaction and cost-efficient implementation. Using a fluorescent DNA dye, the HB196 primers perform efficiently in a qPCR context without the HB503 PCR’s enrichment, as displayed in the LOD calculations and the application to eDNA extracts (Fig 1). The HB503 enrichment extends this utility on both eDNA (Fig 1A) and pure DNA templates (Fig 1B), increasing sensitivity down to 5 copies/µL with 35 cycles of enrichment—with 20 cycles of enrichment striking a more ideal balance between quantification discernment and sensitivity. While enrichment is valuable for more sensitive detection (presence/absence), it can create challenges in the quantification of eDNA samples because preamplification does not always produce linear template enrichment across low copy samples—supported by our observation of greater standard error in the Ct values vs. copy number from HB503-enriched DNA templates (Fig 1B).

The stochastic, exponential nature of PCR—in which reaction efficiency of low-copy targets may be adversely impacted by the presence of inhibitors and concentrations of off-target DNA [65]—renders quantification of eDNA in qPCR uncertain in comparison to ddPCR where the thousands of individual reactions provide a more accurate estimate of starting concentration [69]. This uncertainty in quantification may be further enlarged when employing an enrichment step [65,66]. The enriched nested PCR protocol thus offers improved sensitivity in detection, but less certainty in absolute quantification. We demonstrate the utility of the HB196 primers alone (no enrichment step) in qPCR with SYBR dye for relative quantification and the functionally similar EvaGreen dye (Bio-Rad) in ddPCR to maximize sensitivity. The resultant eDNA concentrations measured in ddPCR (Fig 2B) are a function of a host of variables including the temporal component of the animal’s DNA release, fluid dynamics within the stream, and nucleic acid dispersion, dilution and decay as well as variability between replicates [70,71]. We observed greater variability in *C. alleganiensis* DNA closer to the source in our ddPCR analysis and we hypothesize this to be the result of the aforementioned variables that dilute/disperse eDNA as it travels downstream. Additionally, the dynamic decay process of eDNA in surface waters cannot be ignored [72]. Often, eDNA is used for detection (presence/absence) with additional interest in gleaning potential animal location/abundance information when possible. While location and animal number are not immediately available from quantitative DNA analysis, the correlation to target copy number and signal strength can be helpful in guiding future field tests.

### 4.2. Impacts of off-target amplification on eDNA assays

The CytB104 qPCR method has served as a viable detection assay for *C. alleganiensis* mitochondrial DNA across diverse geographies cited [38–41]. However, instances of unintended amplification [41] combined with our results from Fig 3A indicate that the CytB104 primer set’s off-target amplification has the potential to alter presence/absence results, which would be deleterious for eDNA detection methods critical to population surveys of an endangered animal. It is important for any eDNA assay to be placed in the proper context—where it may serve as an indicator of animal presence, but not an absolute marker, as even dead or displaced animals may leave lingering eDNA. eDNA results often serve to inform field surveys in a complementary manner—ideally providing additional population information that cannot be obtained by traditional methods. The off-target amplification observed using CytB104 is more likely to interfere with qPCR efficiency than to produce a false positive result, since this method relies on a TaqMan probe for detection, and our qPCR results did not show any meaningful fluorescence when tested on all the off-target amplicons, which were purified and used as qPCR template (see Fig S25 in S1 File). However, the possibility remains for a false negative as we demonstrated in Fig 3, if off-target animal/bacterial sources are sufficiently abundant. This potential false negative could prove particularly deleterious for field surveys that don’t rely on any other methods, since animals may go undetected and potentially injured or displaced. Furthermore, it is also possible for these off-target amplicons to generate a false positive via conventional PCR, especially if they are observed at the correct length (such as in Fig 3B, Lane 1), if there is no follow up DNA sequencing of the amplicon.

### 4.3. Considerations for HB503/HB196 primer sets in future eDNA applications

Table 1 shows the high specificity of the HB503 and HB196 primer sets in comparison with the equivalent sequences of *N.* maculosus and *D. fuscus*—two salamanders known to reside in adjacent Appalachian habitats with meaningful homology in cytochrome b genes. The nested primers target a region of the D-loop that differs substantially between salamander species—with 4–14 base pair mismatches among the three species considered—while the CytB104 primers rely on 2–5 base pairs of differentiation to prevent primers from binding. Considering our off-target analysis of CytB104 showing amplification of the *N. maculosus* equivalent, 100 bp, cytochrome b region (Fig 3B, Lane 1), and the HB503/HB196 sets’ proven consistency on the same templates (see Figs S7, S17 in S1 File), the nested set’s improved resolution between salamander species appears to be critical in avoiding false positive amplification.

As eDNA research continues to improve animal detection in aquatic systems, nucleic acid amplification accompanied by DNA sequencing—either through Sanger or high-throughput sequencing (HTS)—will take on a growing role in habitat surveillance and conservation. The nested PCR method presented here demonstrates sensitivity which should prove valuable in future eDNA detection studies, such as those that leverage HTS for mtDNA hypervariable region sequencing analysis. The D-loop’s intraspecific sequence diversity uniquely allows for examination of the most genetically diverse region of mtDNA—a capability that may bolster future eDNA studies in examining population dynamics with greater precision. Similarly, future work that combines the method we have described here with total nucleic acid extractions (instead of DNA alone) and a subsequent comparison of DNA/RNA targets may enable more in-depth analyses concerning animal range, gene expression, and biological activity in surface waters.

## Supporting information

S1 File Supplemental results and methods. (DOCX)

S2 File HB503 forward primer BLASTn results. (CSV)

S3 File HB503 reverse primer BLASTn results. (CSV)

S4 File HB196 forward primer BLASTn results. (CSV)

S5 File HB196 reverse primer BLASTn results. (CSV)

S1 Raw Images Unedited DNA gel electrophoresis images. (PDF)
