# Supplementary material for: Nested PCR amplification of the mitochondrial hypervariable region for non-invasive eDNA detection of Cryptobranchus alleganiensis
Source: PLoS One. 2025 Jul 23;20(7):e0328633. doi: 10.1371/journal.pone.0328633 (PMC12286349; doi:10.1371/journal.pone.0328633)
Supplement: S1 File — (DOCX) [file pone.0328633.s001.docx]

**Supplemental Figures**

All DNA Agarose Electrophoresis Analysis was performed on SYBR 2% E-Gel with Invitrogen TrackIt™ 1kbp Plus DNA ladder. Images processed with Adobe Photoshop.


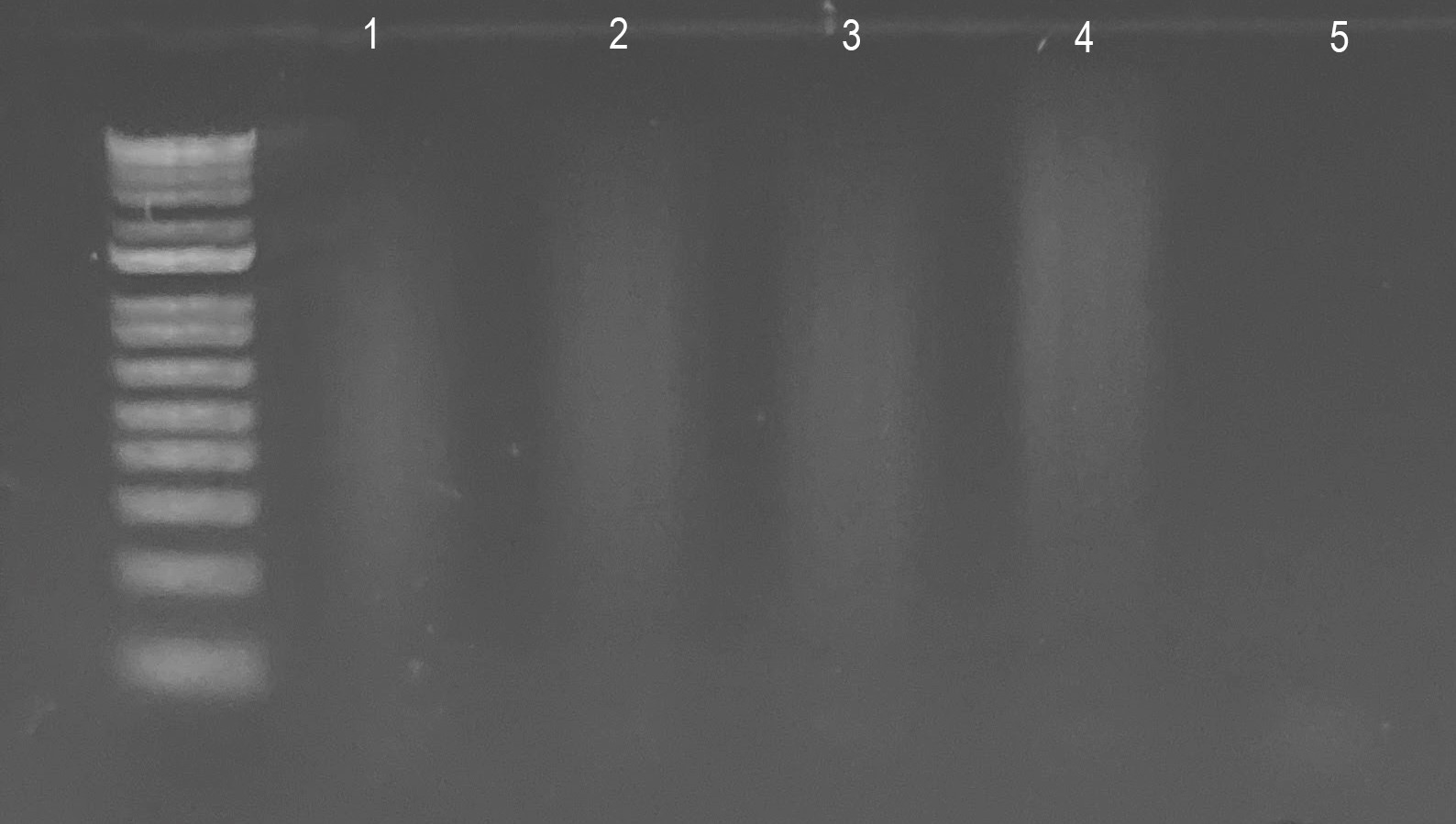


Figure S1. HB503 PCRs on *C. alleganiensis* swabs


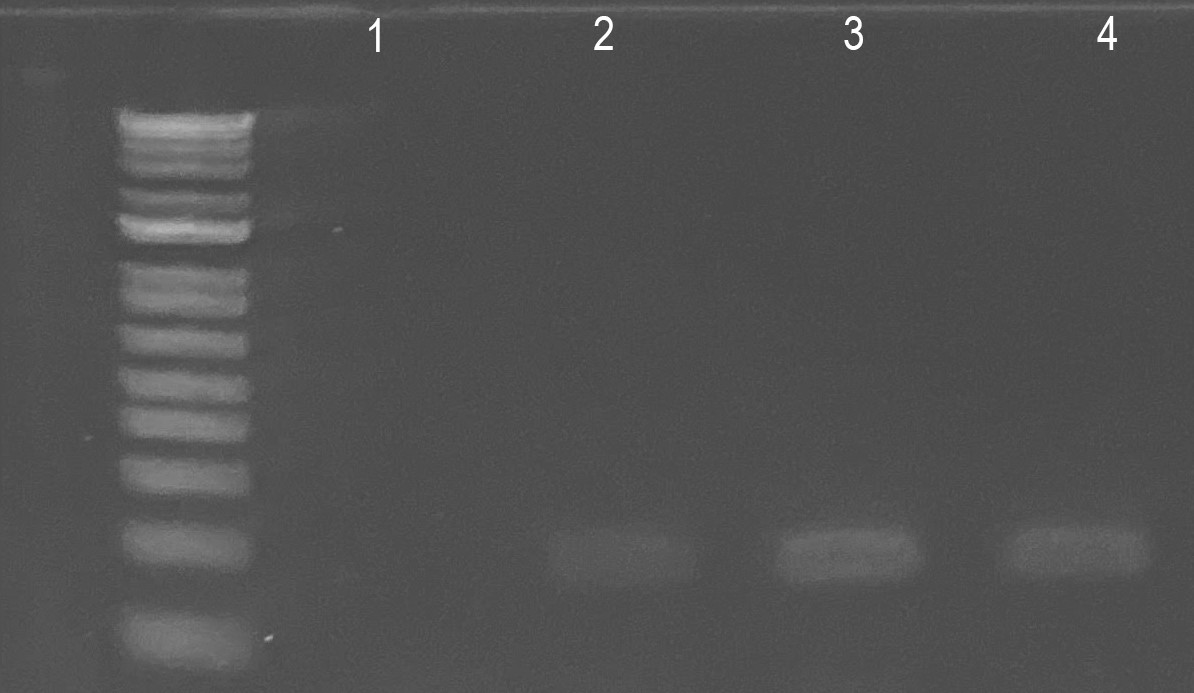


Figure S2. HB196 PCRs on *C. alleganiensis* swabs


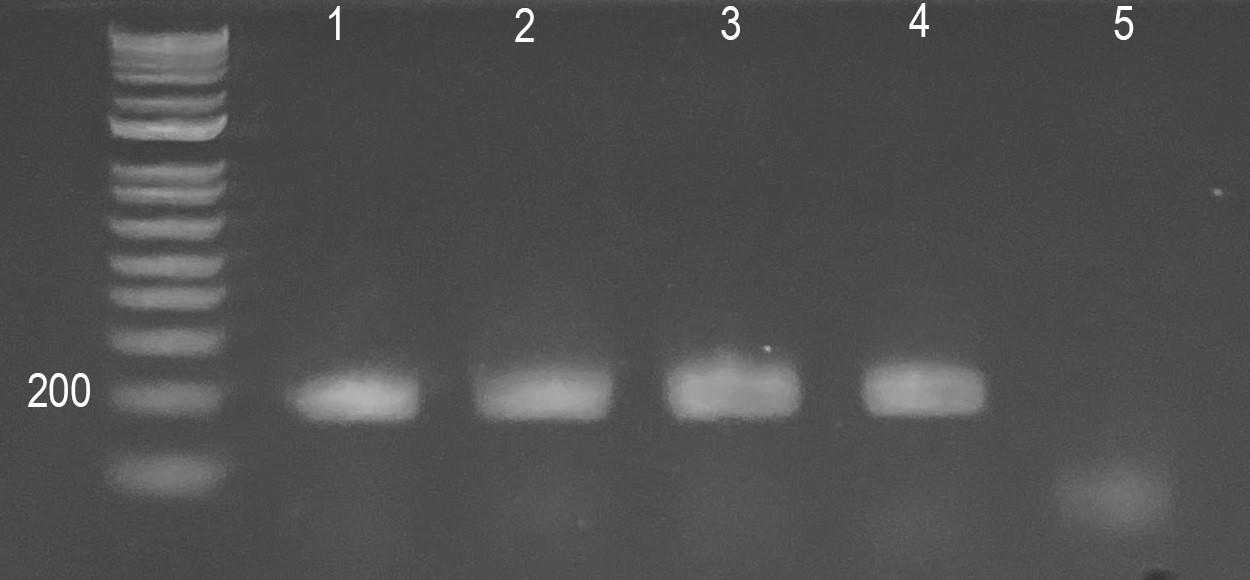


Figure S3. HB196 primers-qPCR E-gel confirmation: dilutions 50,000 copies/µL-50 copies/µL (Lanes 1-4, respectively), Lane 5 NTC, (see Main Figure 2B)


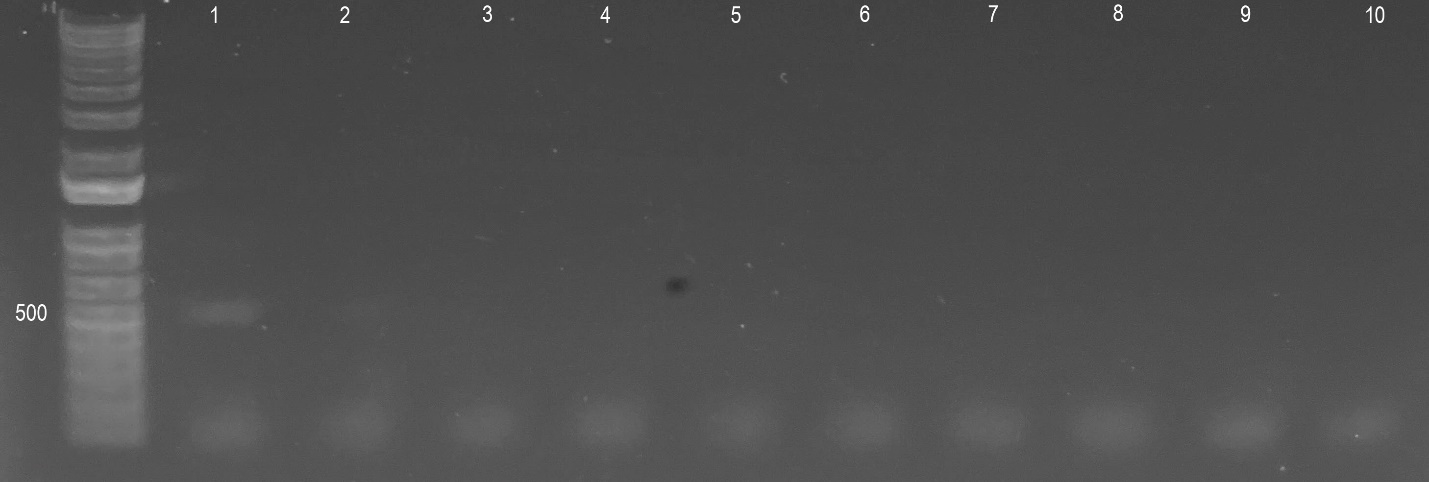


Figure S4. HB503 PCRs on S-Creek eDNA template dilutions .2 ng/µL-2e-8 ng/µL, Lane 9-10 NTC (see Supp. Method 4.1)


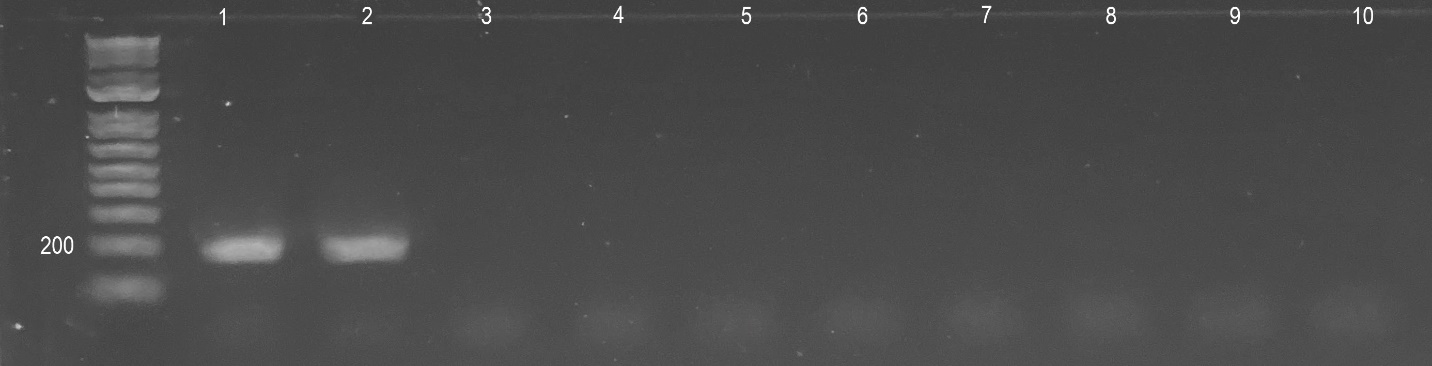


Figure S5. HB196 PCRs on HB503 enriched S-Creek DNA templates (HB503 product diluted 1:10)


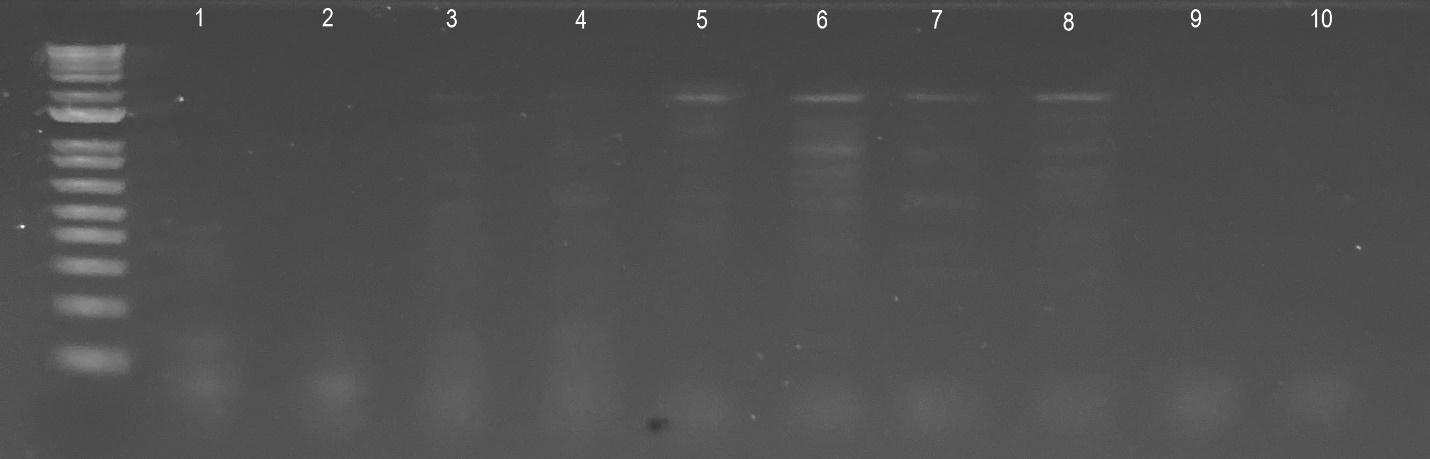


Figure S6. HB503 PCR on mudpuppy DNA templates, Lanes 9-10 NTC


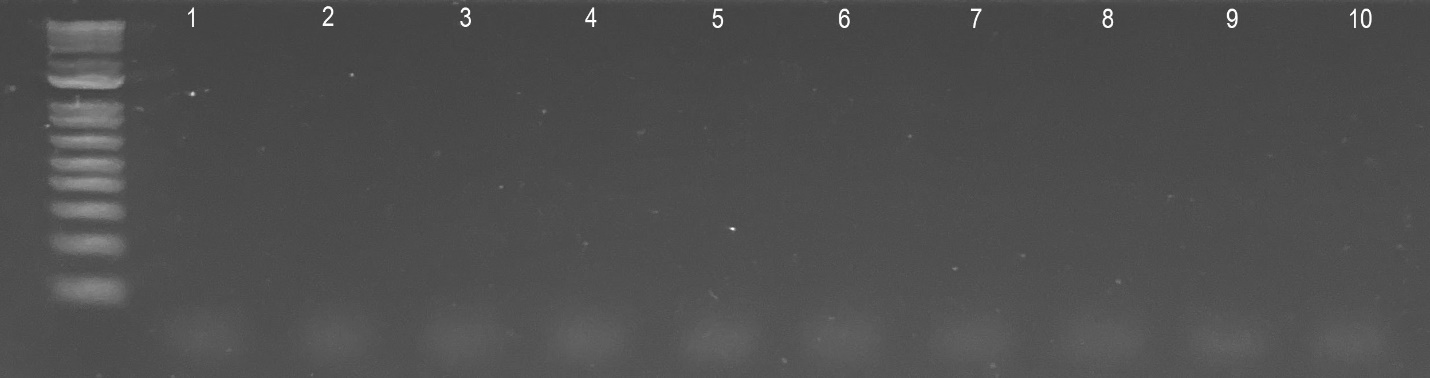


Figure S7. HB196 PCR on HB503 enriched mudpuppy DNA templates, Lanes 9-10 NTC


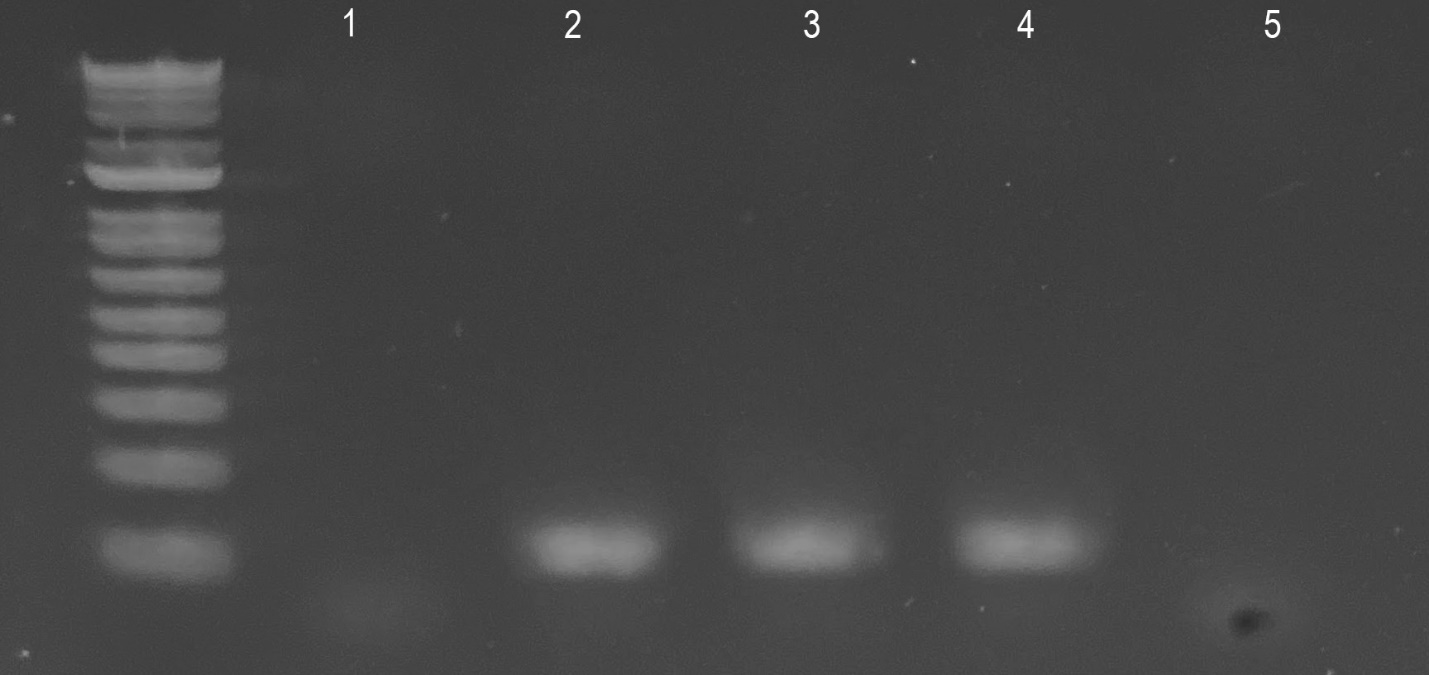


Figure S8. CytB104 PCRs on *C. alleganiensis* swabs


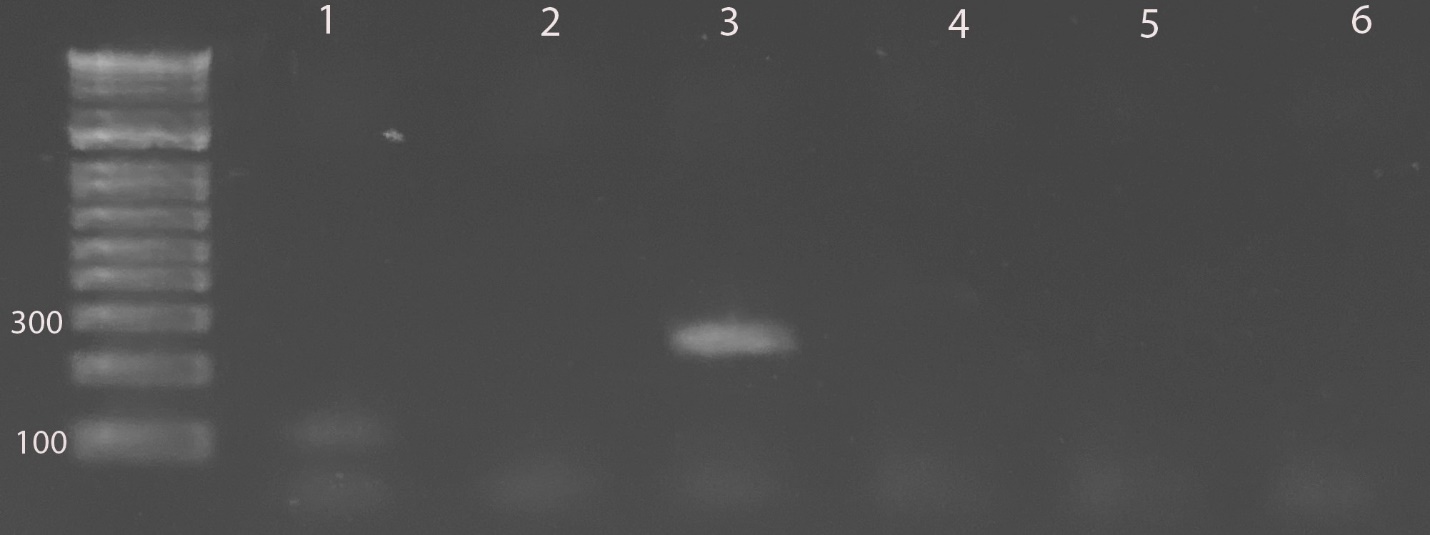


Figure S9. CytB104 PCR on mudpuppy templates, Lanes 5-6 NTC


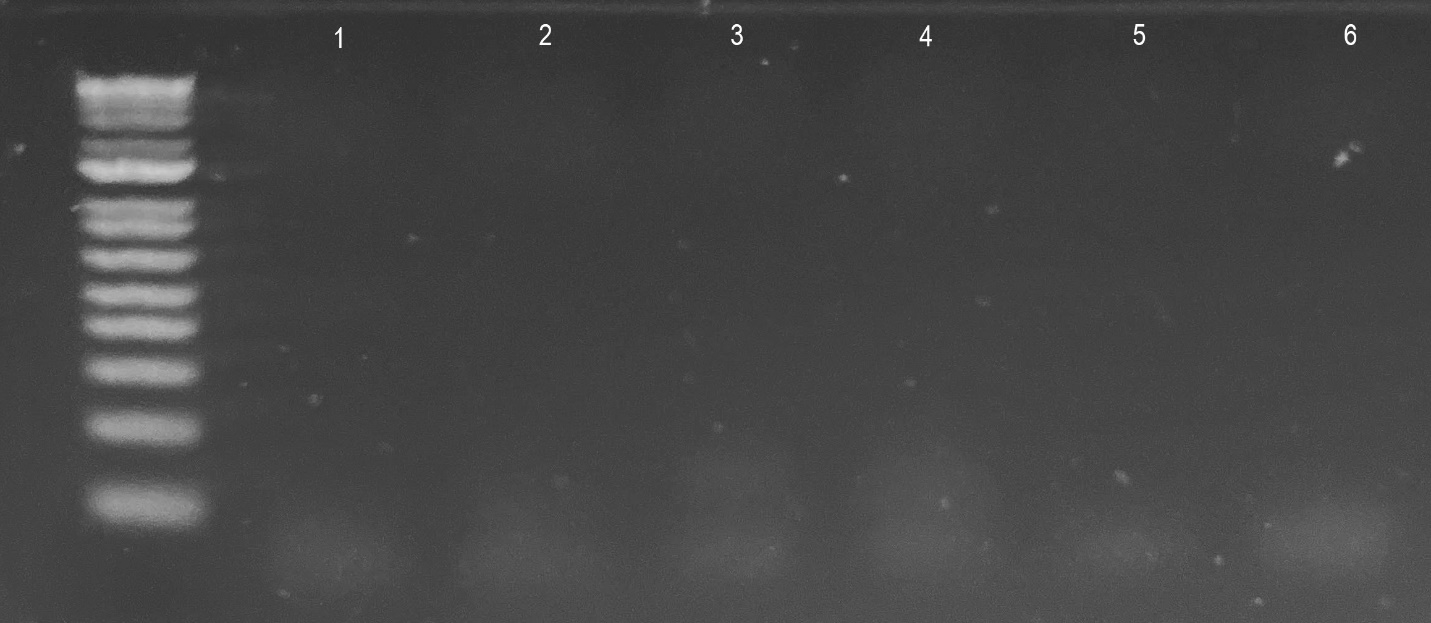


Figure S10. CytB104 PCR on mudpuppy templates, Lanes 5-6 NTC


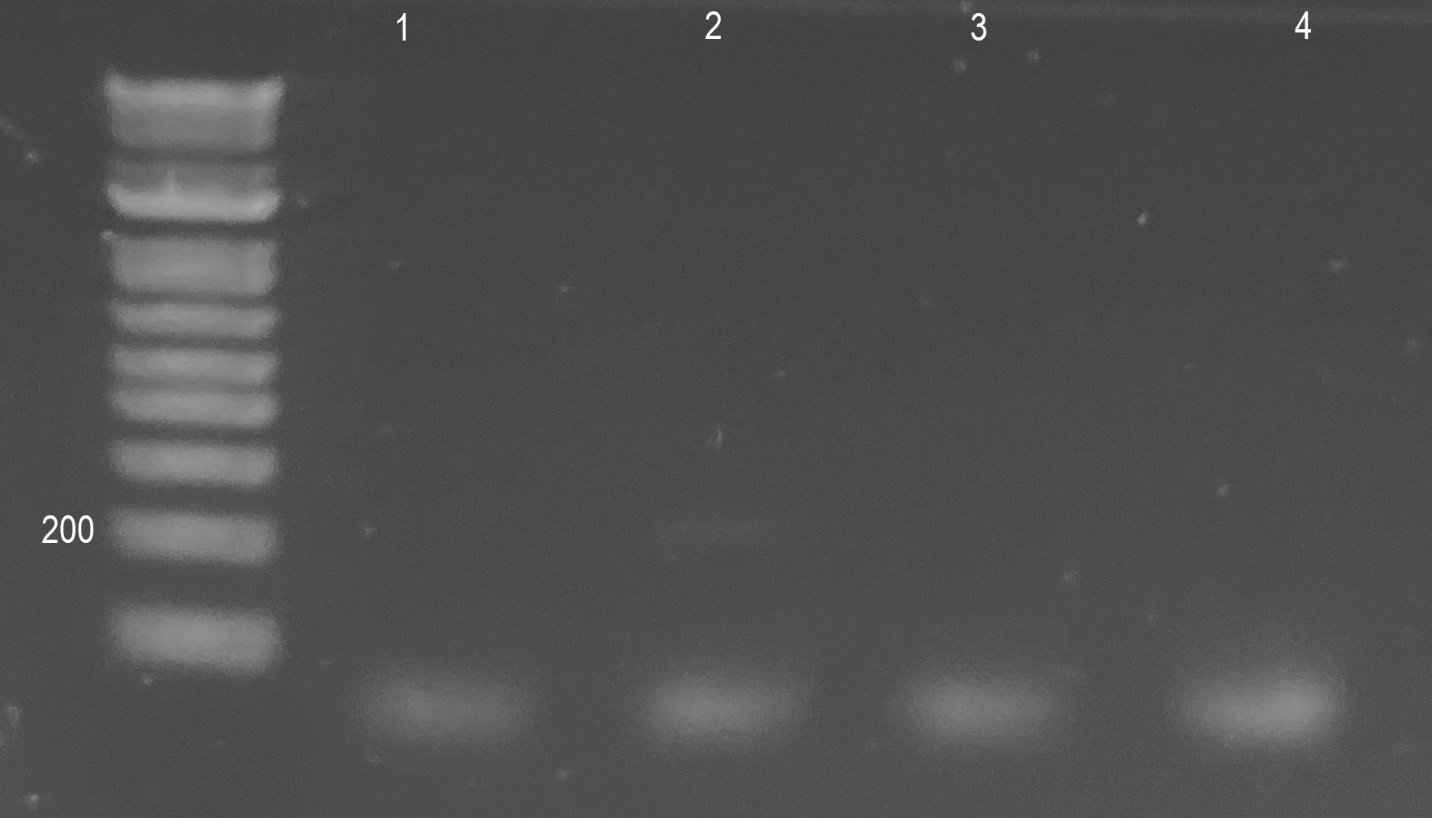


Figure S11. CytB104 PCR on eDNA templates from Little Darby Creek 2024, Lane 4 NTC


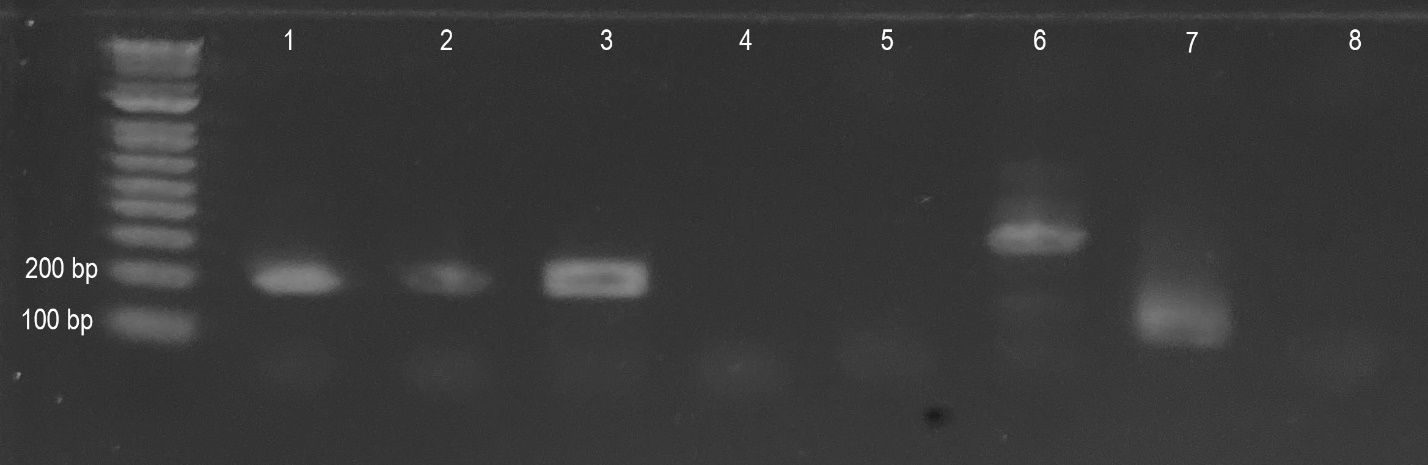
Figure S12. S-Creek eDNA templates, Lanes 1-2 HB196 PCR on 0 and 800 ft extractions, Lane 4 HB196 positive control, Lane 5 HB196 NTC, Lane 6-7 CytB104 PCR on 0 and 800 ft S-Creek extractions, Lane 8 CytB104 positive control, Lane 9 CytB104 NTC


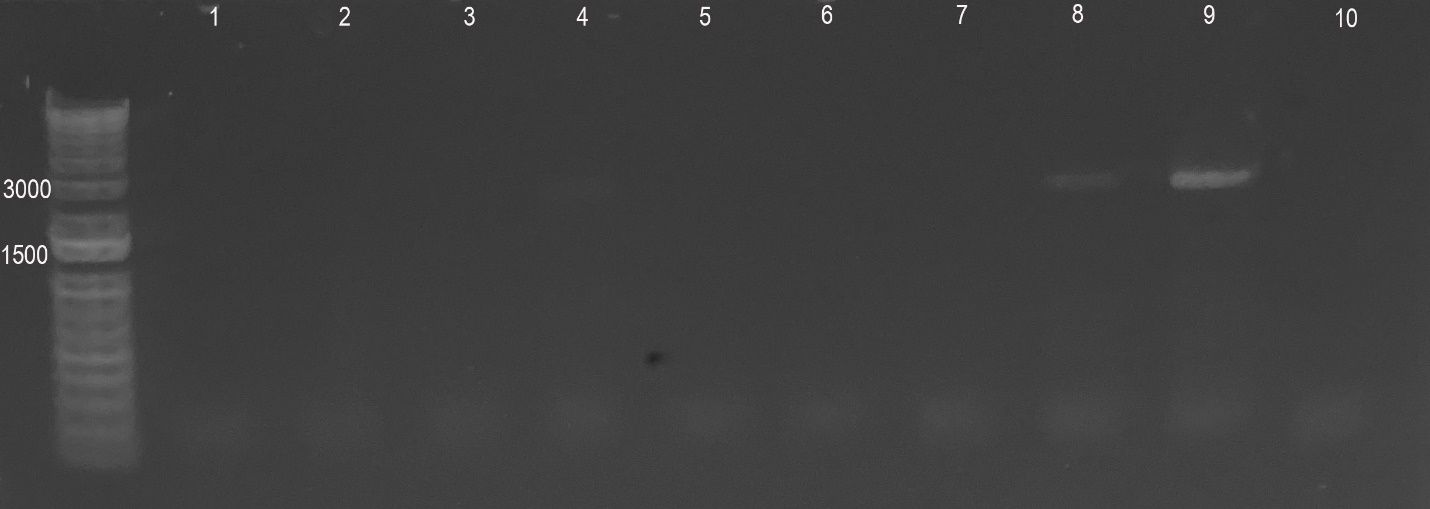


Figure S13 Mudpuppy Control Region PCRs (Lanes 1-9 Individual mudpuppy DNA extraction templates, Lane 10 NTC)


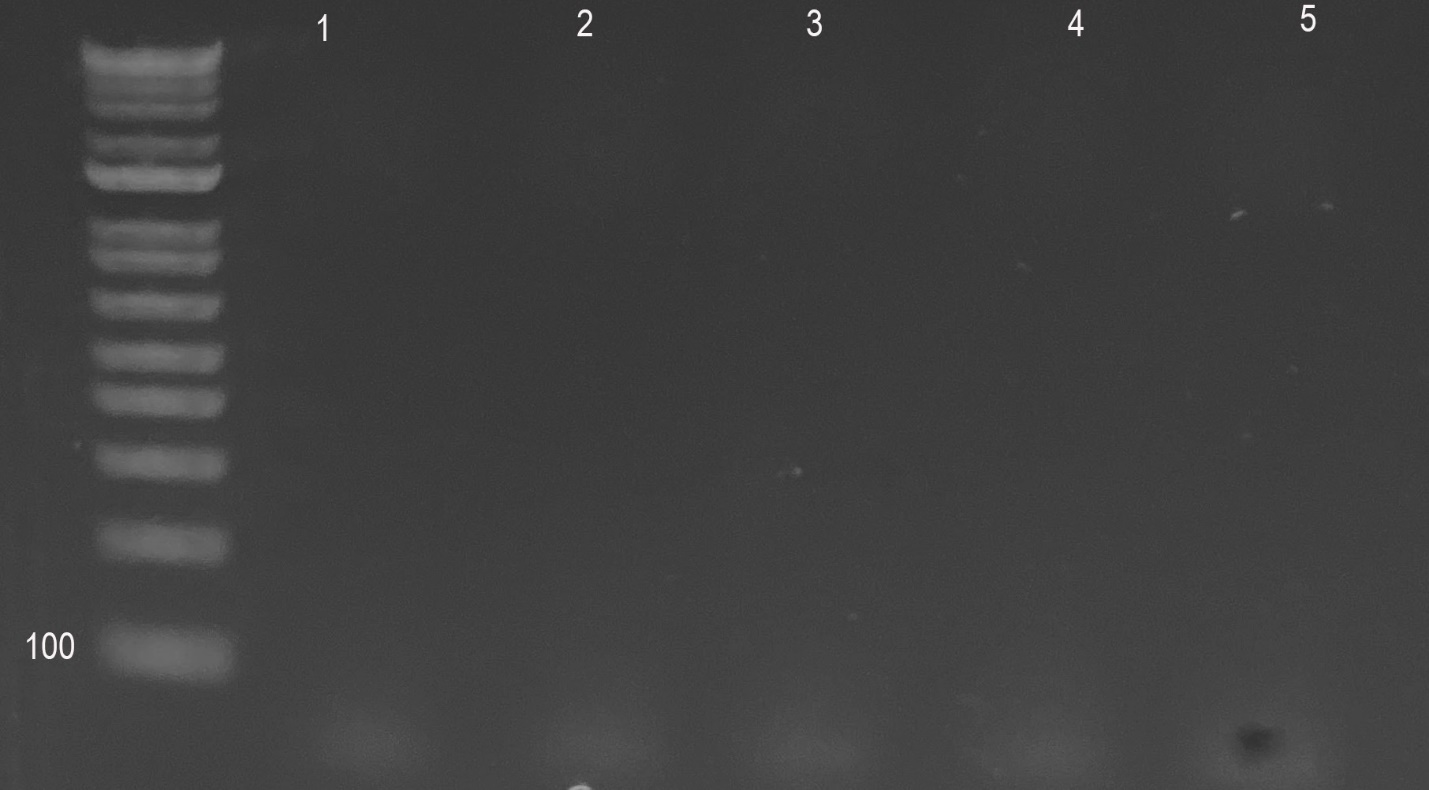


Figure S14 CytB104 PCR results on *D. fuscus* DNA template*, Lanes 4-5 NTC


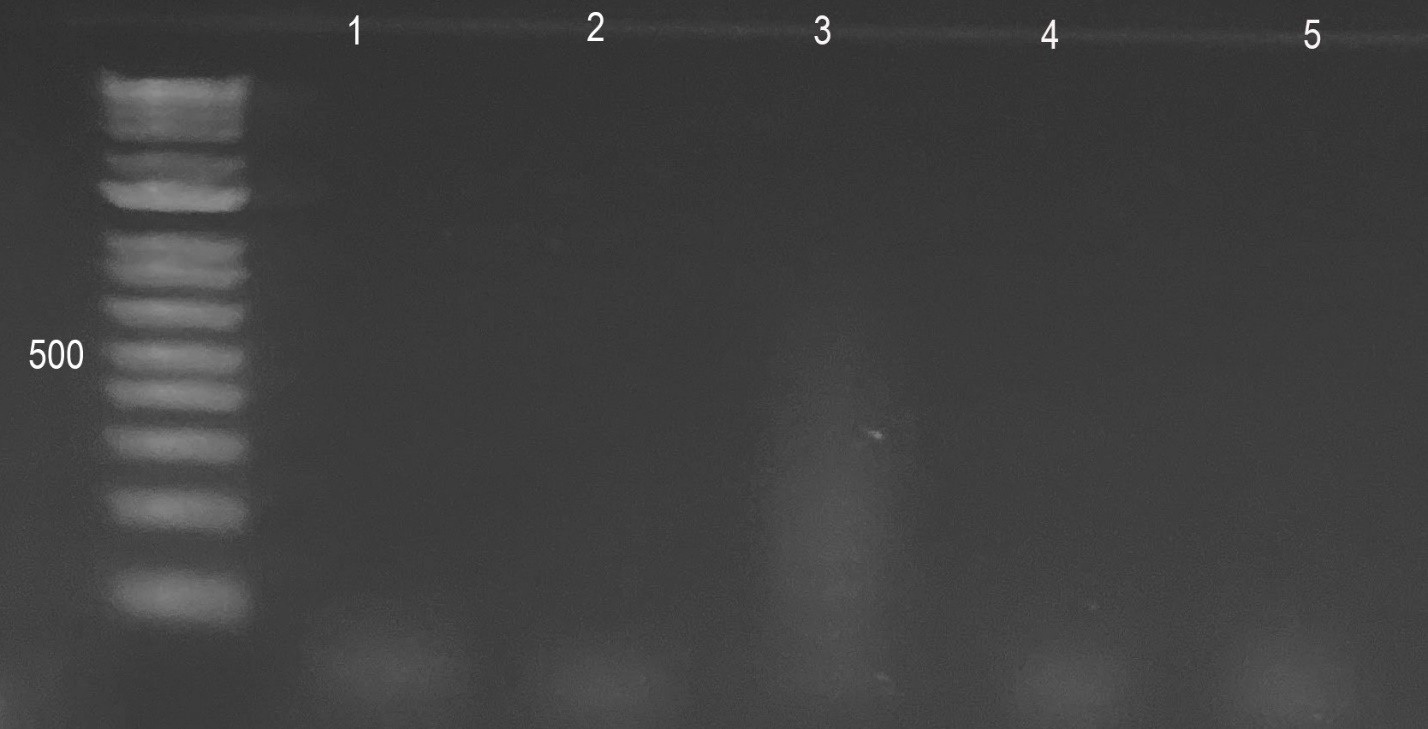


Figure S15 503 PCR results on *D. fuscus* DNA template*, Lanes 4-5 NTC


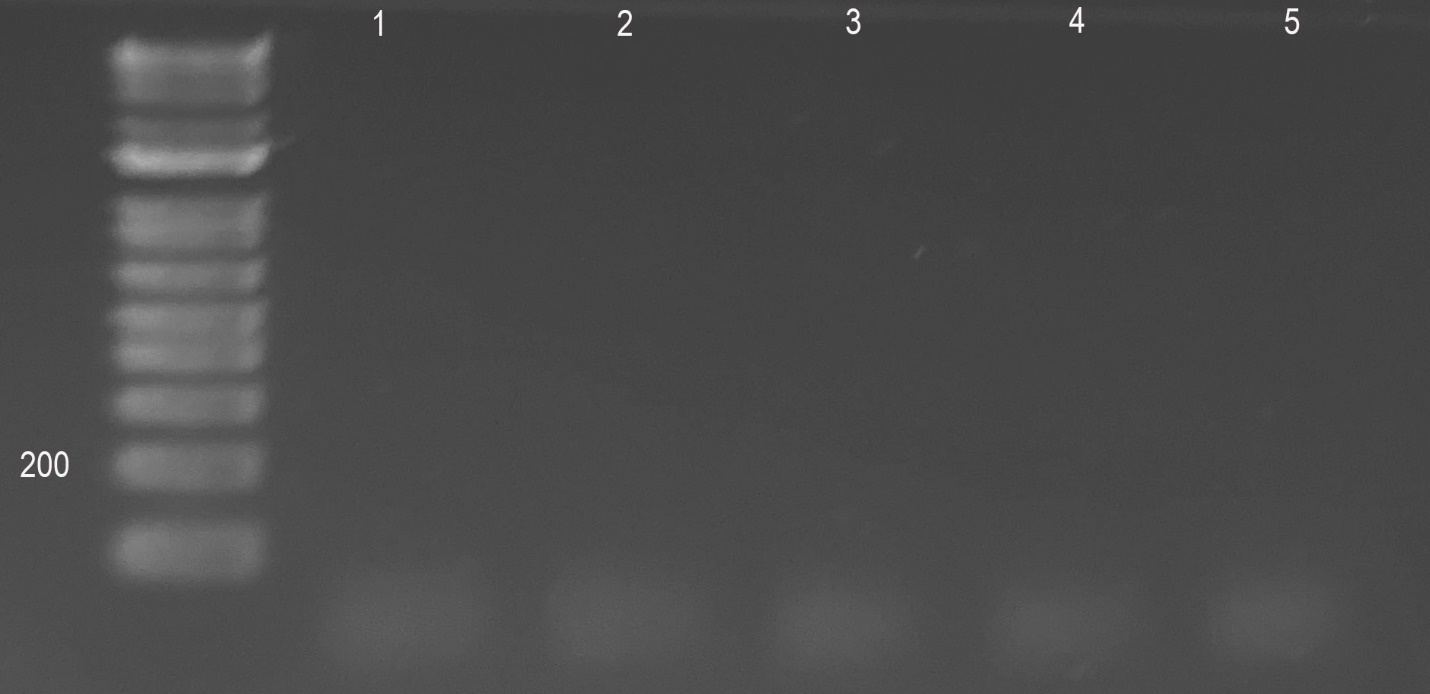


Figure S16 Nested PCR results on 503 PCR-enriched *D. fuscus* DNA templates*, Lanes 4-5 NTC

*Accession Information: Northern Dusky Salamander

RAP3641 GSU-27996 5 November 2024 adult Virginia Powhatan


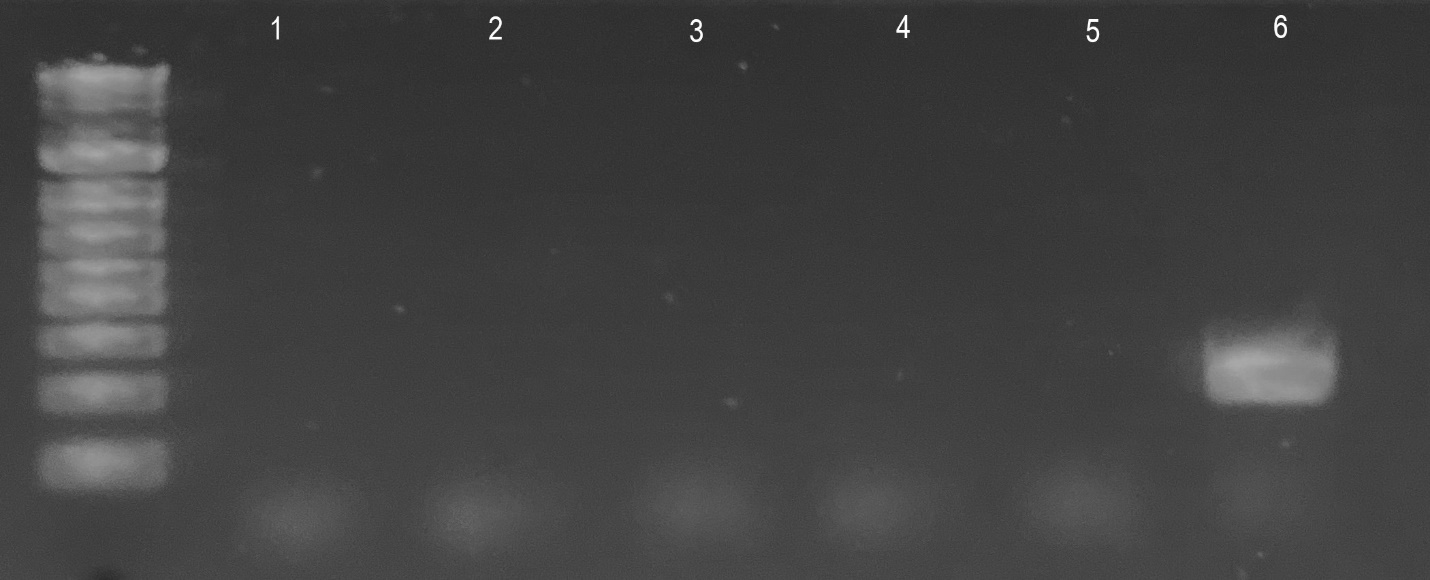


Figure S17 HB196 PCR results directly on mudpuppy DNA templates (no HB503 enrichment), Lane 10 NTC, Lane 11 positive control


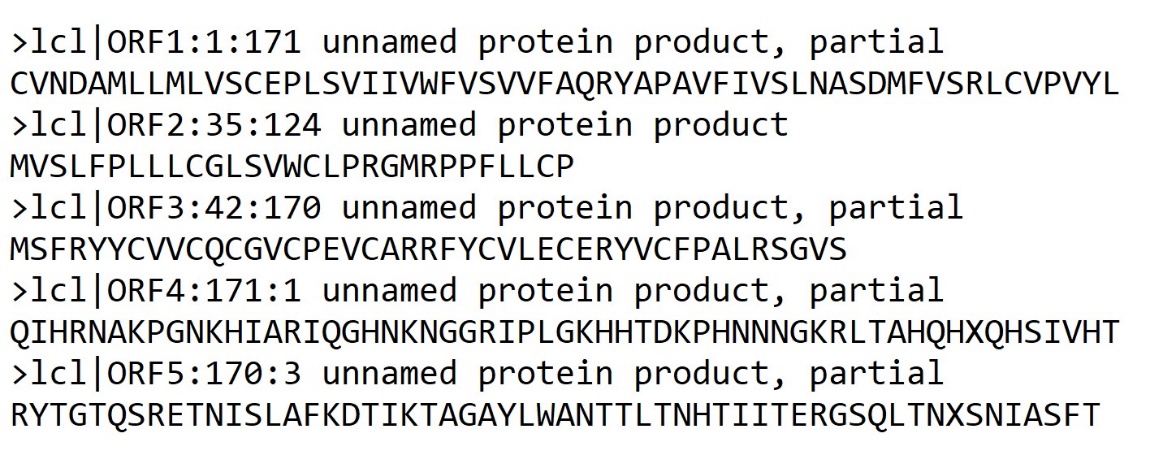


Figure S18 CytB104 Little Darby Creek Off-Target Amplicon (Fig. S11 Lane 2): ORFfinder results


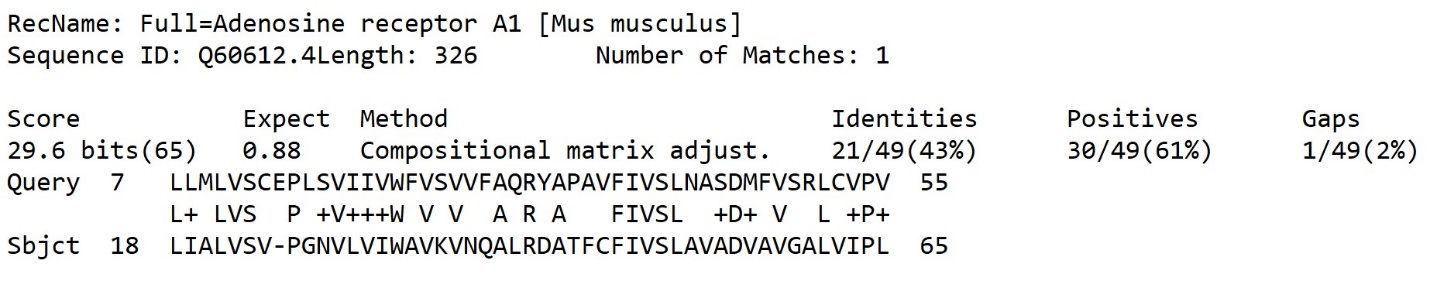


Figure S19. CytB104 Little Darby Creek Off-Target amplicon: ORF1 BLASTp result


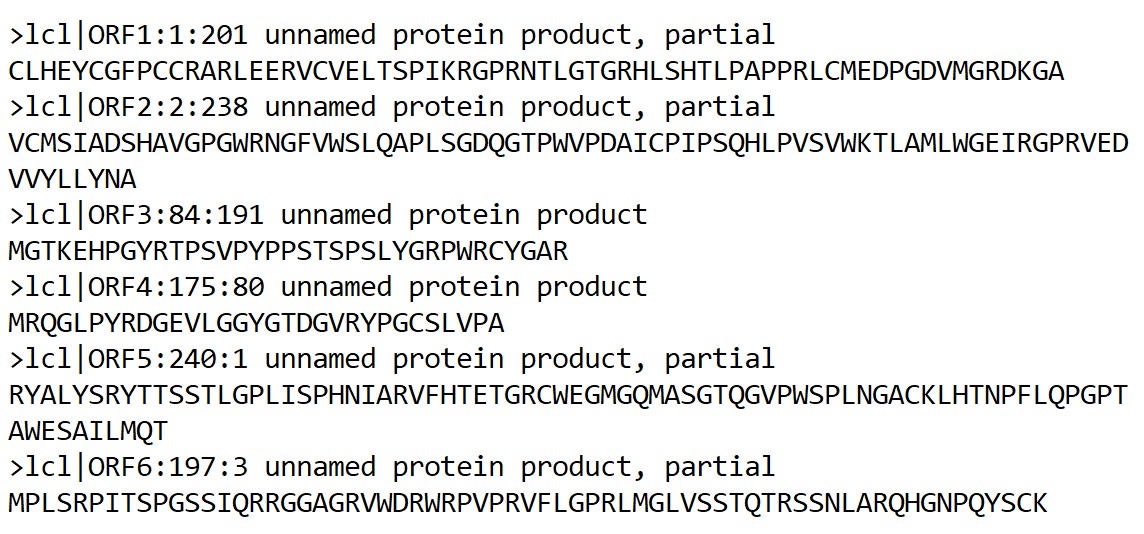


Figure S20. CytB104 Mudpuppy Template Off-Target Amplicon: ORFfinder results


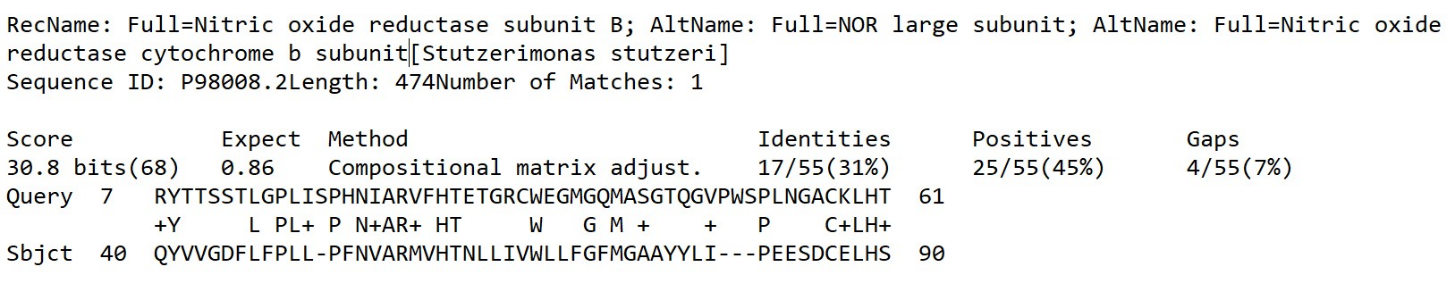


Figure S21. CytB104 Mudpuppy Template Off-Target Amplicon: ORF5 BLASTp Results


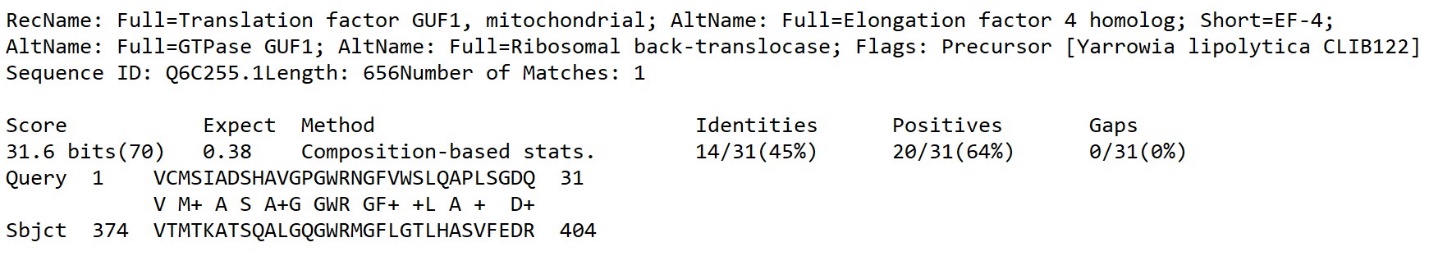


Figure S22. CytB104 Mudpuppy Template Off-Target Amplicon: ORF2 BLASTp Results


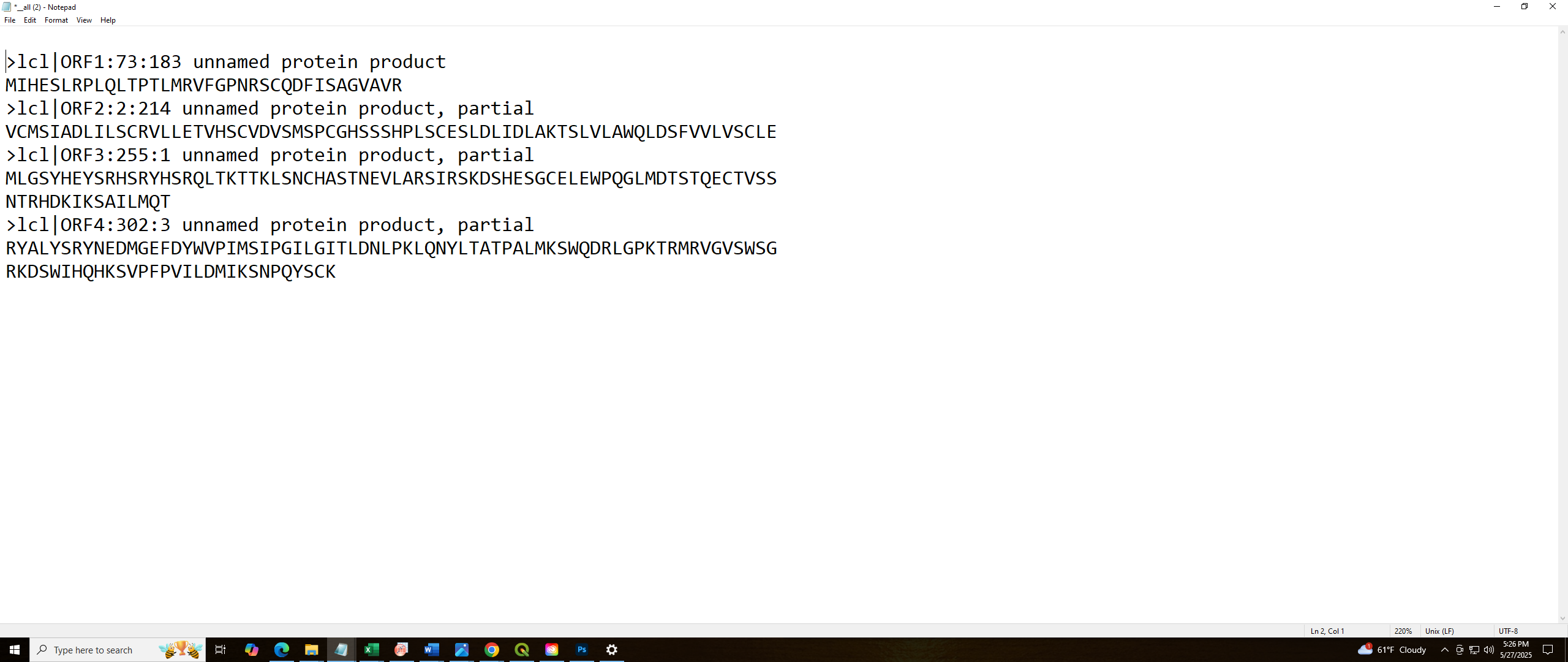


Figure S23. CytB104 S-Creek Amplicon: ORFfinder Results


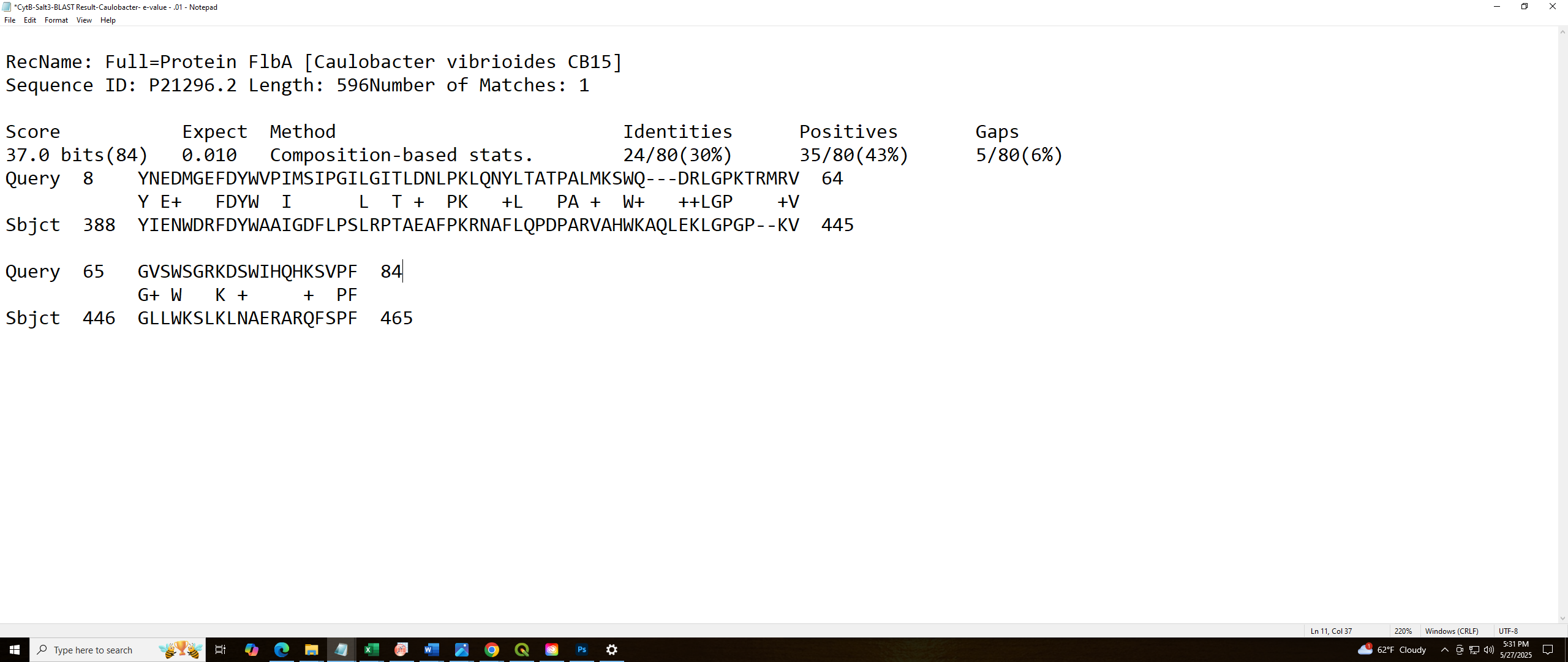


Figure S24. CytB104 S-Creek Off-target amplicon: ORF4 BLASTp Results


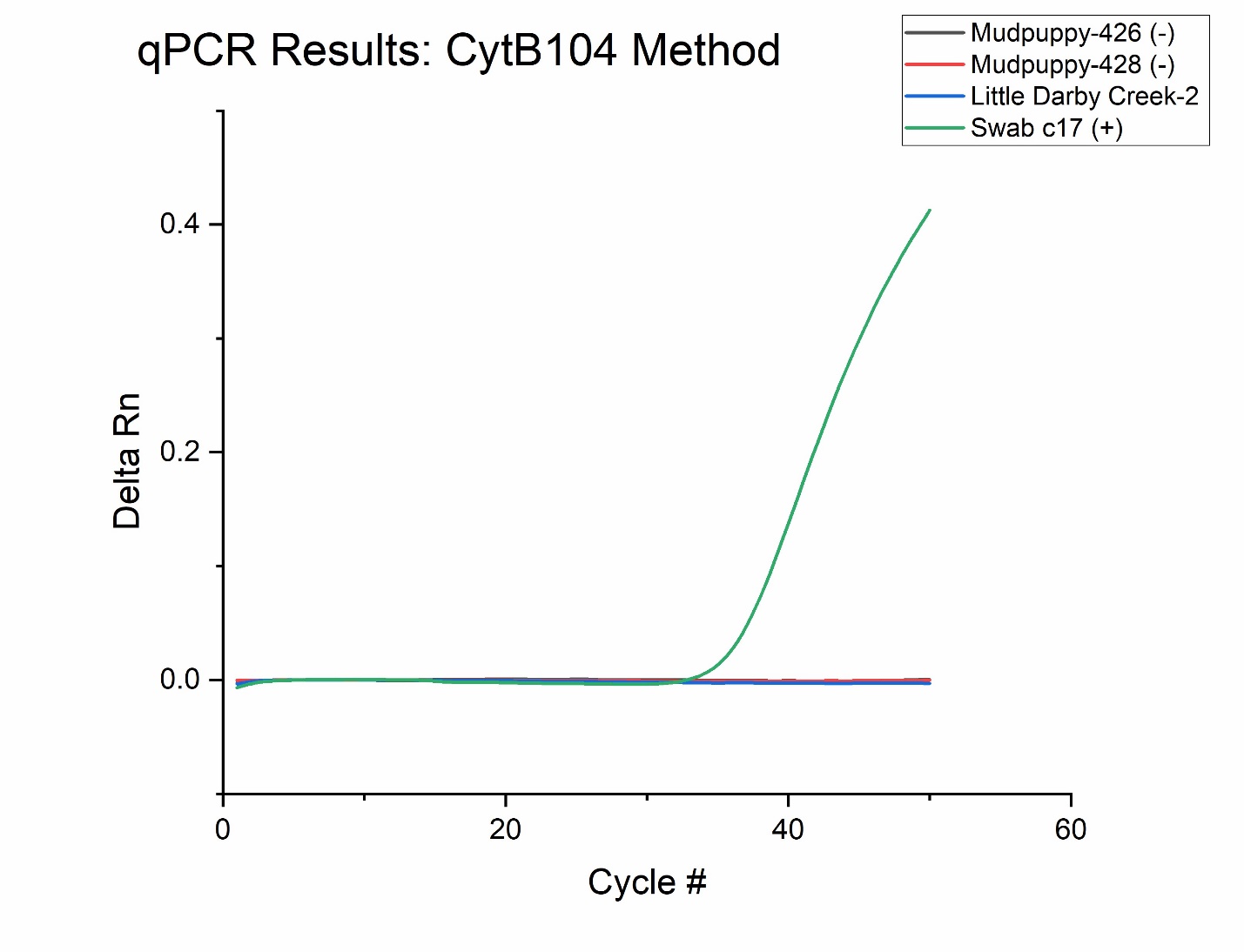


Figure S25 qPCR Results: CytB104 Method on mudpuppy templates 426, 428; eDNA template Little Darby Creek #2; *C. alleganiensis* swab c17


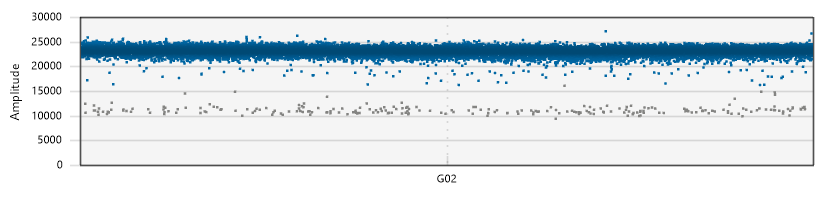
Figure S26. ddPCR result on a positive control swab using HB503/HB196 primer protocols (see main Methods 2.3). Threshold was implemented based on BioRad’s instructions

**Supplemental Methods**

Method S1.

Conventional PCR Limit of Detection: HB503/HB196 Nested PCR Method

A limit of detection for eDNA samples was established for the HB503-HB196 primer sets by serial diluting a positive eDNA sample (S-Creek 1) in nuclease free water (Invitrogen). HB503 PCRs were completed according to the standard method on template dilutions down to 2 x 10-8 ng/µL. The HB503 PCRs were then DNA purified using Qiagen MinElute, diluted to approximately .5 ng/µL, and used as template for subsequent Nested PCRs. (S4-S5)

Method S2.

Mudpuppy Control Region PCRs and Sequencing

Primer walking via Sanger Sequencing was completed by firstly designing primers within a conserved region of the 12s rRNA and Cytochrome b genes:

Mudpuppy-12s Reverse: 5’ GTAGCTCGTAGTACTCTGGCGAATA 3’

Mudpuppy-CytB Forward: 5’ GCATATTGGACGAGGCTTATATTATGGTT 3’

Using the first round of Sanger sequencing results, additional primers were designed based on consensus sequences to elucidate the entire control region. These sequencing primers are as follows:

12sMud-Seq Reverse: 5’ TAAGGCTAGGACCAAACCTTTATGT 3’

CR-Mud-Seq Forward: 5’ TAATACTCTTATCTTGACCTGAATCGGA 3’
