# Supplementary material for: Nested PCR amplification of the mitochondrial hypervariable region for non-invasive eDNA detection of Cryptobranchus alleganiensis
Source: PLoS One. 2025 Jul 23;20(7):e0328633. doi: 10.1371/journal.pone.0328633 (PMC12286349; doi:10.1371/journal.pone.0328633)
Supplement: S1 Raw Images — (PDF) [file pone.0328633.s006.pdf]

File S1: Unedited Gel Electrophoresis Pictures and Additional Supplemental Information

All DNA Agarose Electrophoresis Analysis was performed on SYBR 2% E-Gel with Invitrogen TrackIt™ 1kbp Plus DNA ladder.

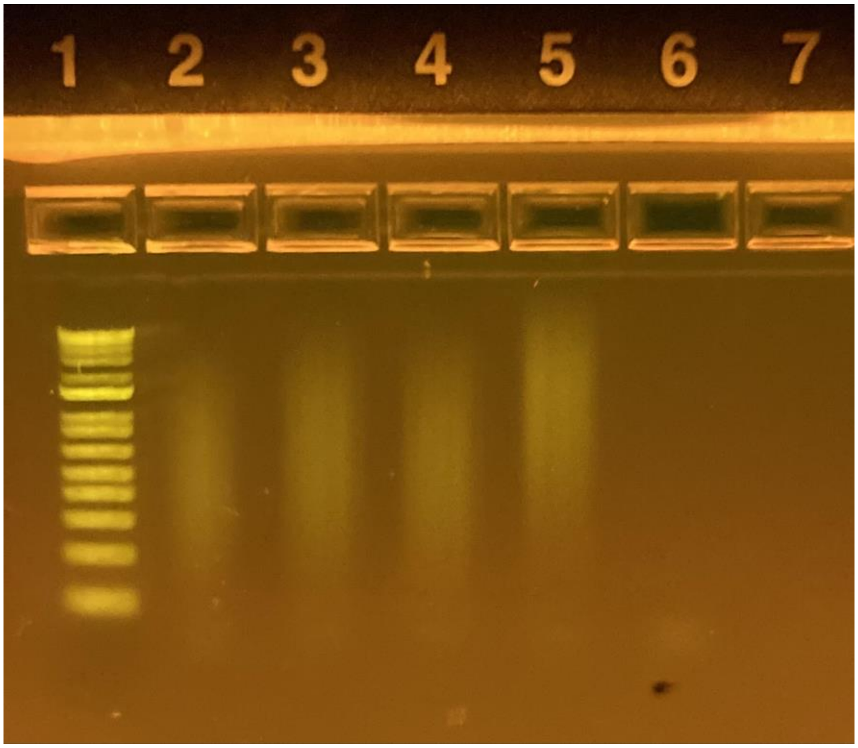

Figure S1. HB503 PCRs on *C. alleganiensis* swabs

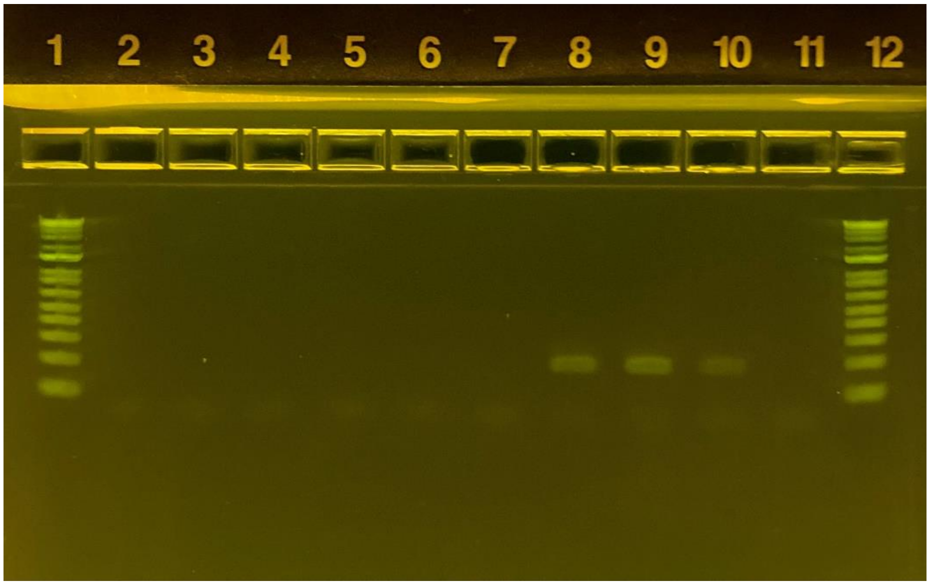

Figure S2. HB196 PCRs on *C. alleganiensis* swabs

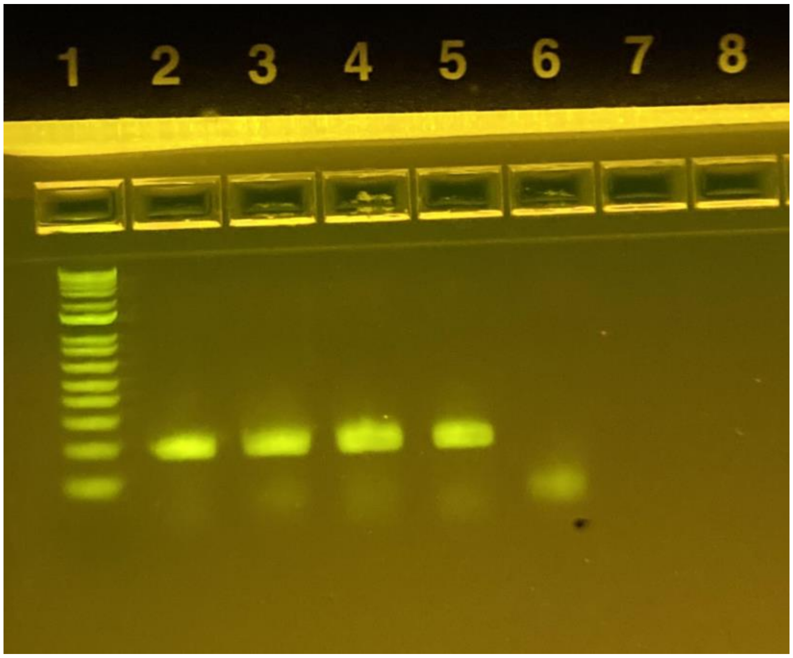

Figure S3. HB196 primers-qPCR E-gel confirmation: dilutions 50,000 copies/µL-50 copies/µL (Lanes 1-4, respectively), Lane 5 NTC, (see Main Figure 2B)

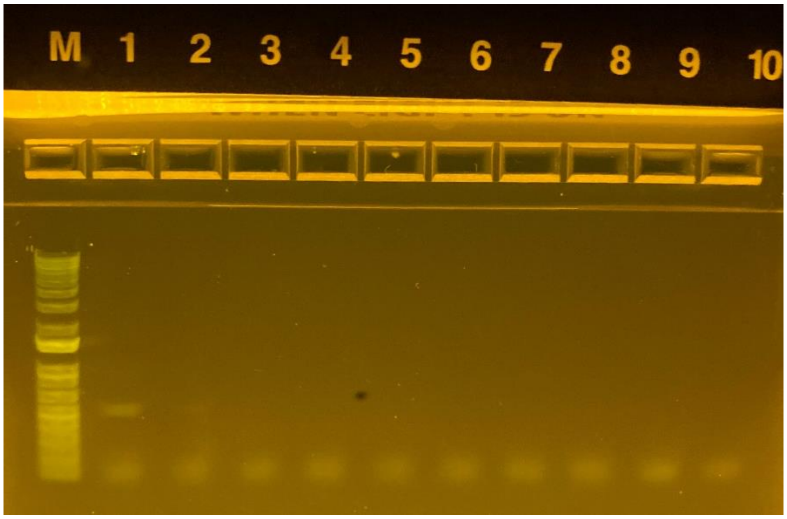

Figure S4. HB503 PCRs on S-Creek eDNA template dilutions .2 ng/µL-2e-8 ng/µL, Lane 9-10 NTC (see Supp. Method 4.1)

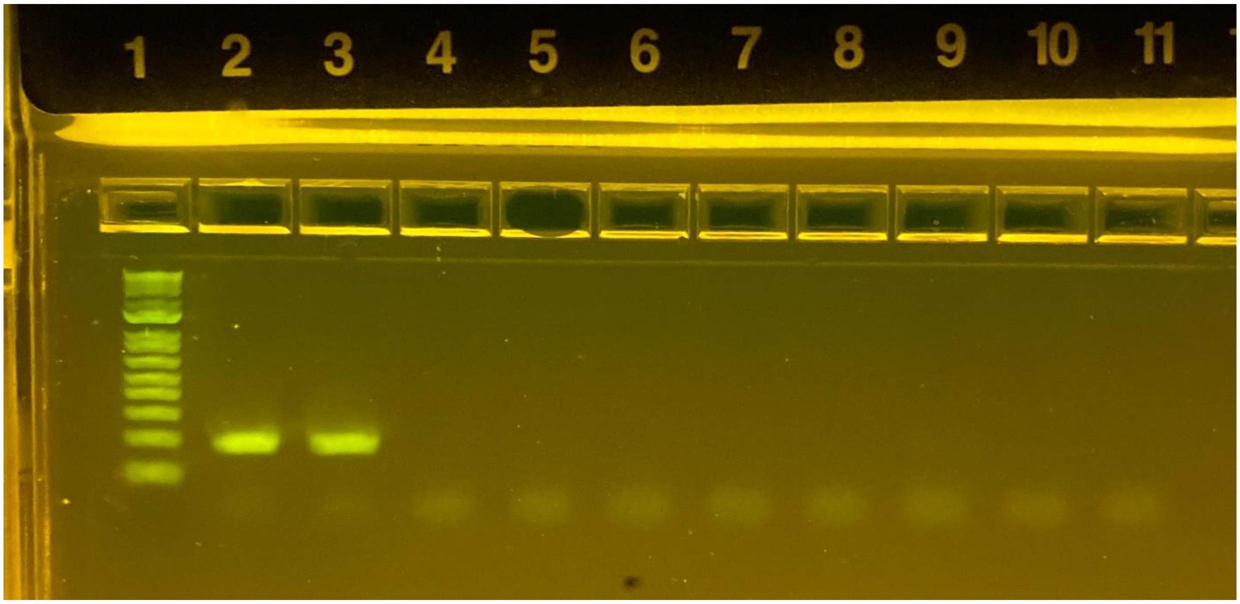

Figure S5. HB196 PCRs on HB503 enriched S-Creek DNA templates (HB503 product diluted 1:10)

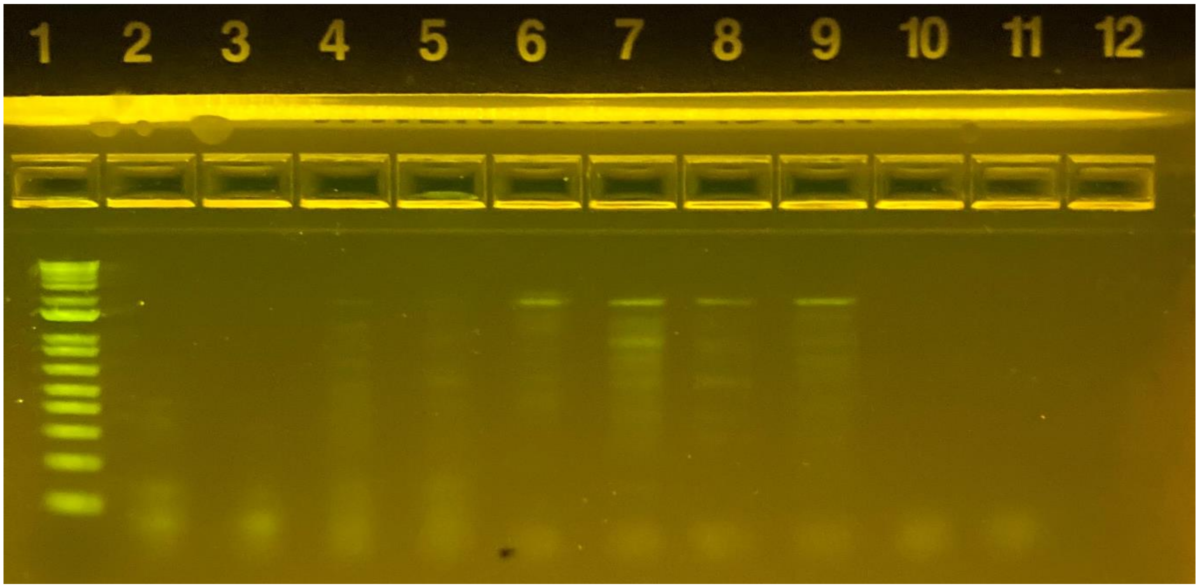

Figure S6. HB503 PCR on *N. maculosus* DNA templates, Lanes 9-10 NTC

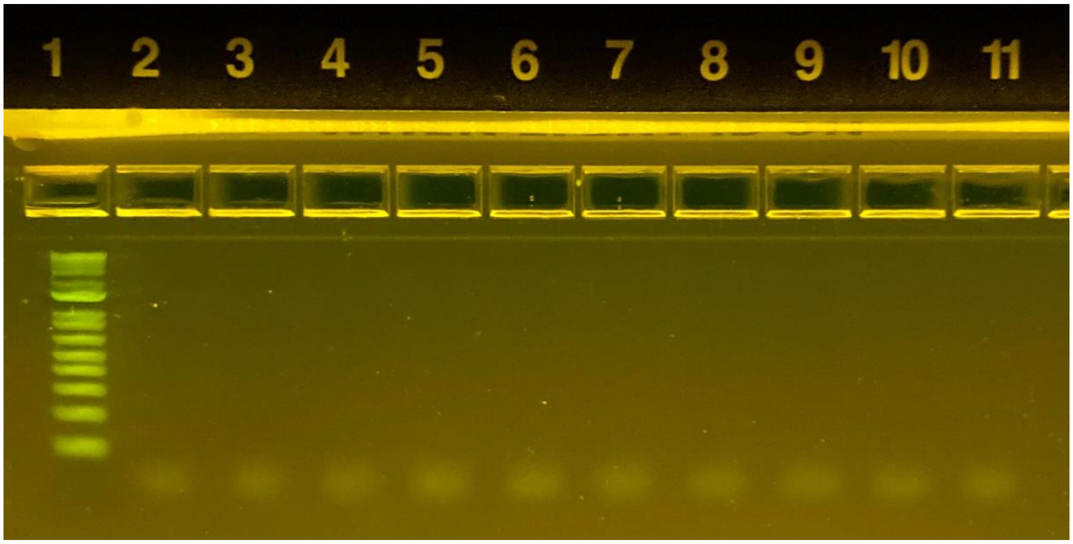

Figure S7. HB196 PCR on HB503 enriched *N. maculosus* DNA templates, Lanes 10-11 NTC

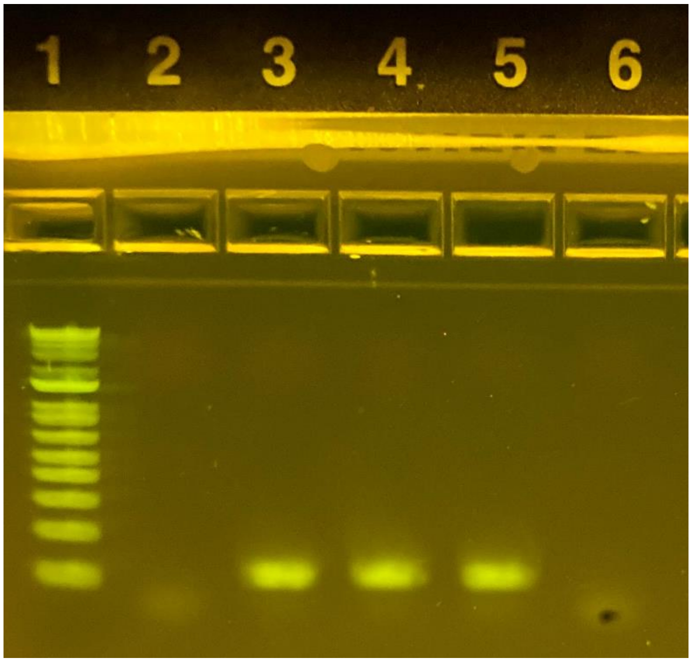

Figure S8. CytB104 PCRs on *C. alleganiensis* swabs, Lane 6 NTC

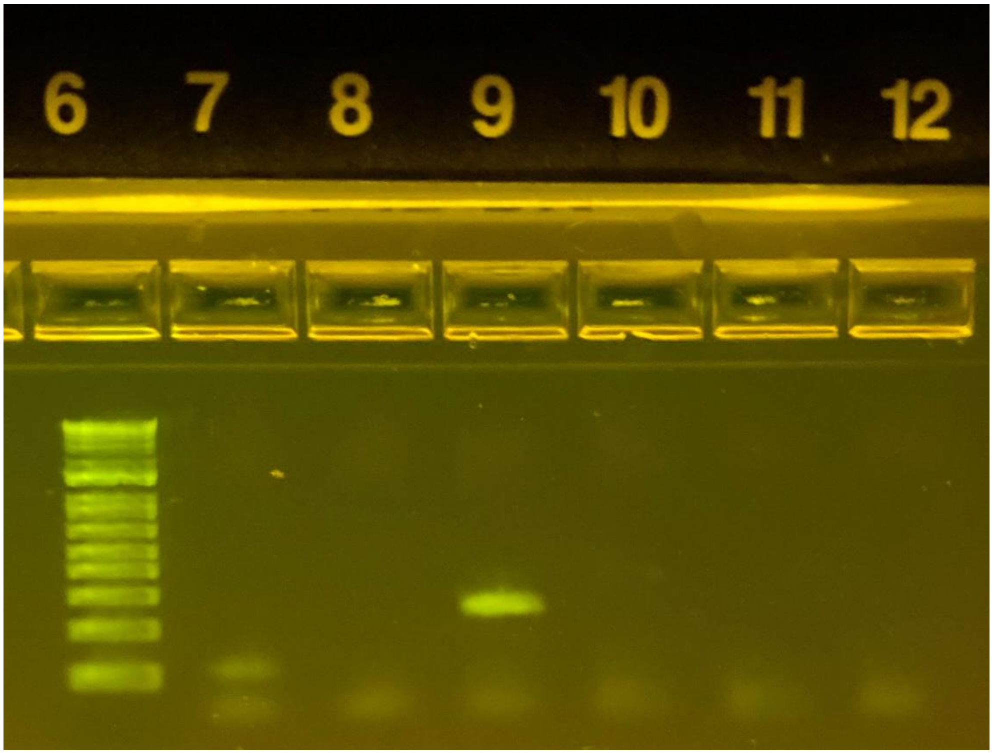

Figure S9. CytB104 PCR on *N. maculosus* templates, Lanes 11-12 NTC

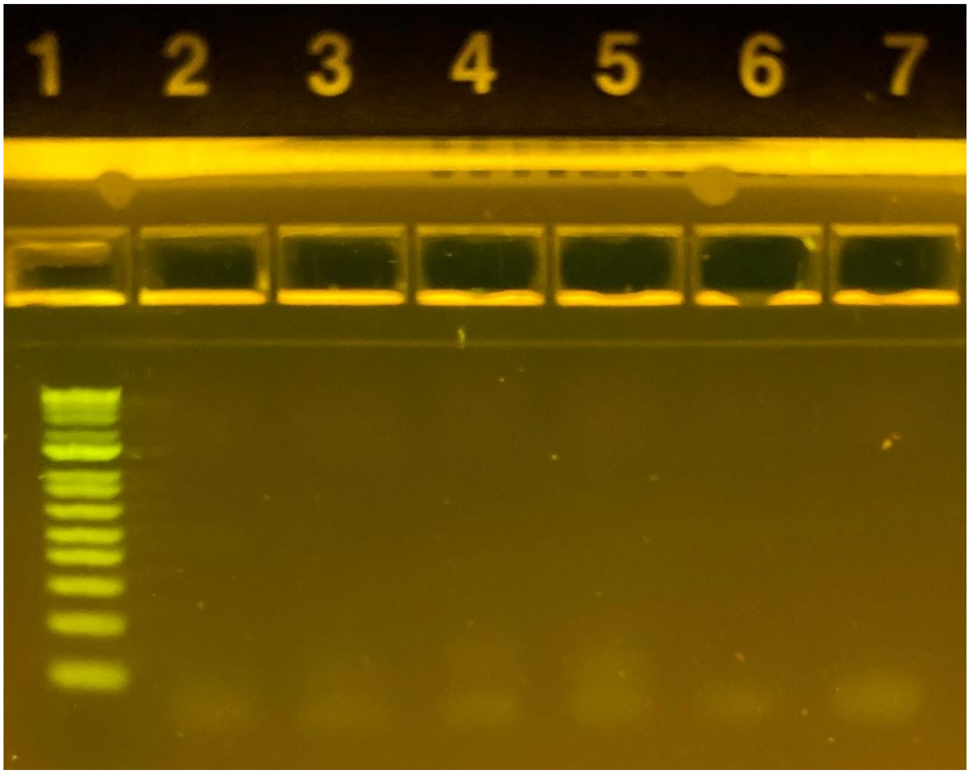

Figure S10. CytB104 PCRs on *N. maculosus* DNA templates, Lanes 6-7 NTC

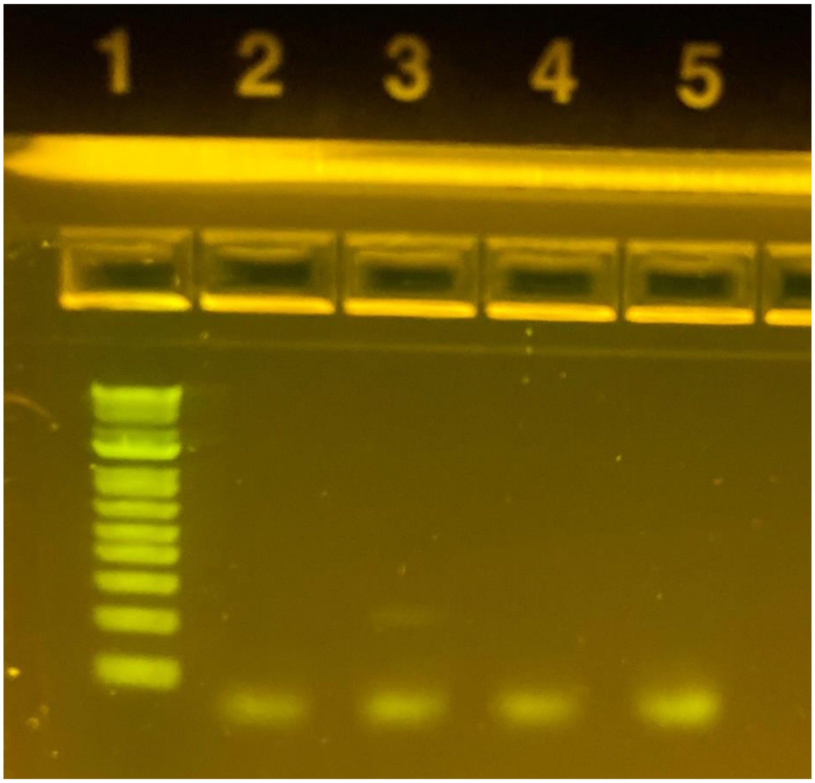

Figure S11. CytB104 PCR on eDNA templates from Little Darby Creek 2024, Lane 5 NTC

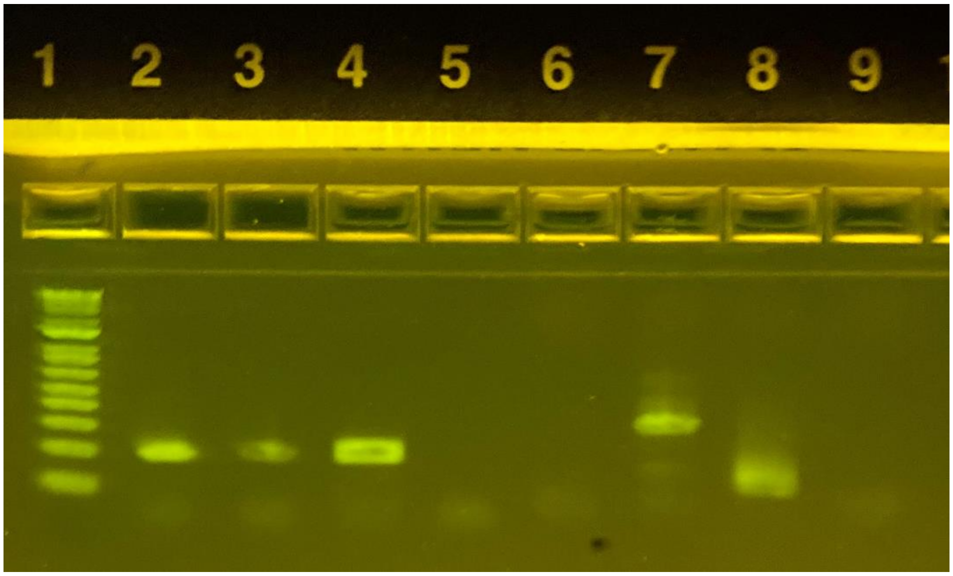

Figure S12. S-Creek eDNA templates, Lanes 2-3 HB196 PCR an 0 and 800 ft extractions, Lane 4 HB196 positive control, Lane 5 HB196 NTC, Lane 6-7 CytB104

PCR on 0 and 800 ft S-Creek extractions, Lane 8 CytB104 positive control, Lane 9 CytB104 NTC

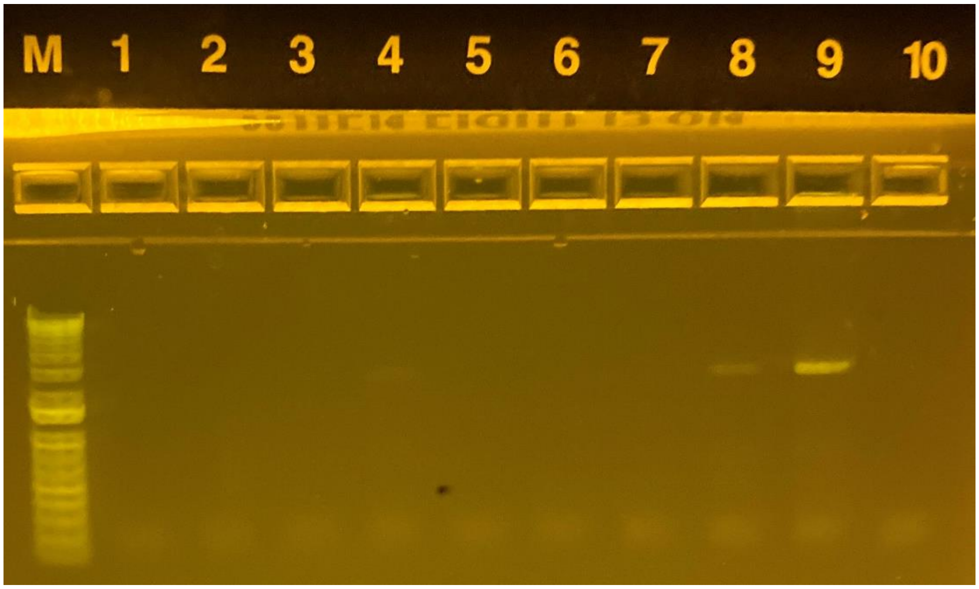

Figure S13. *N. maculosus* Control Region PCRs (Lanes 1-9 Individual mudpuppy DNA extraction templates, Lane 10 NTC)

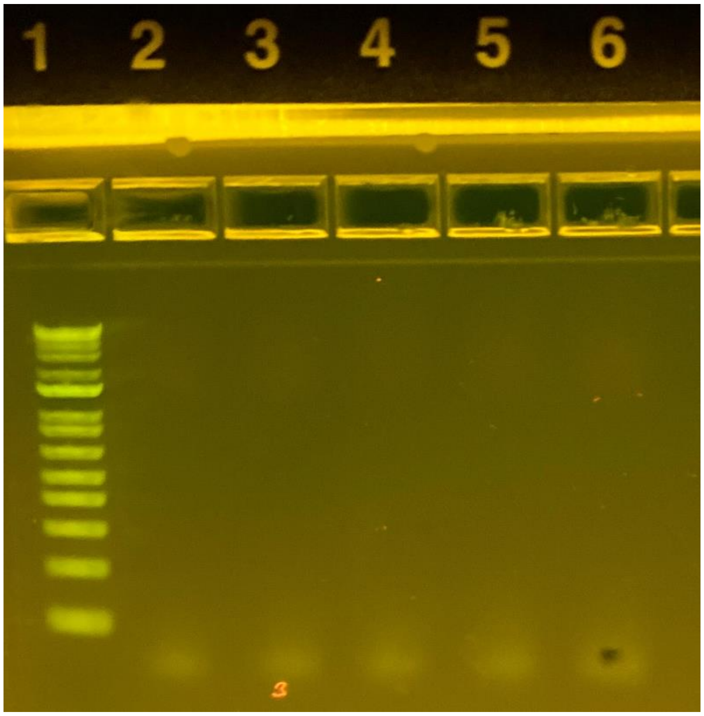

Figure S14. CytB104 PCR results on *D. fuscus* DNA template*, Lanes 5-6 NTC

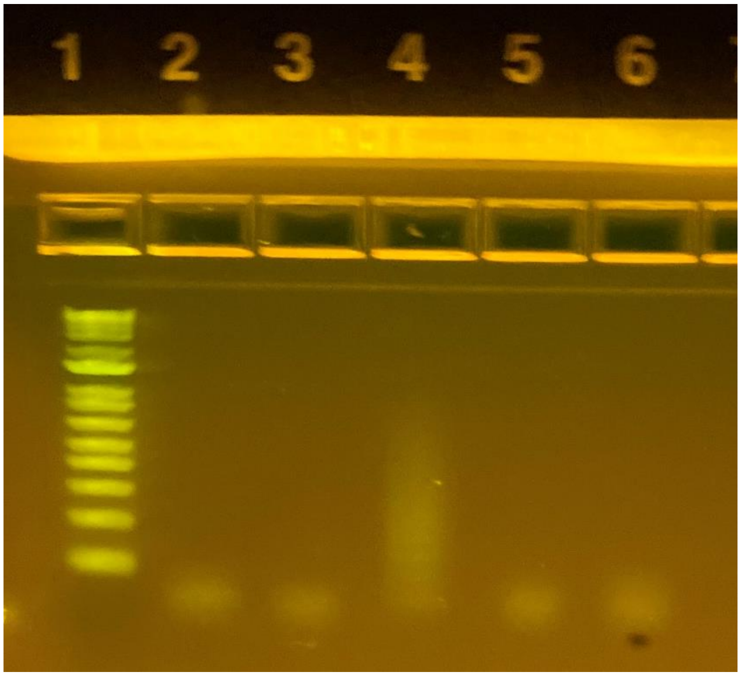

Figure S15. HB503 PCR results on *D. fuscus* DNA template*, Lanes 5-6 NTC

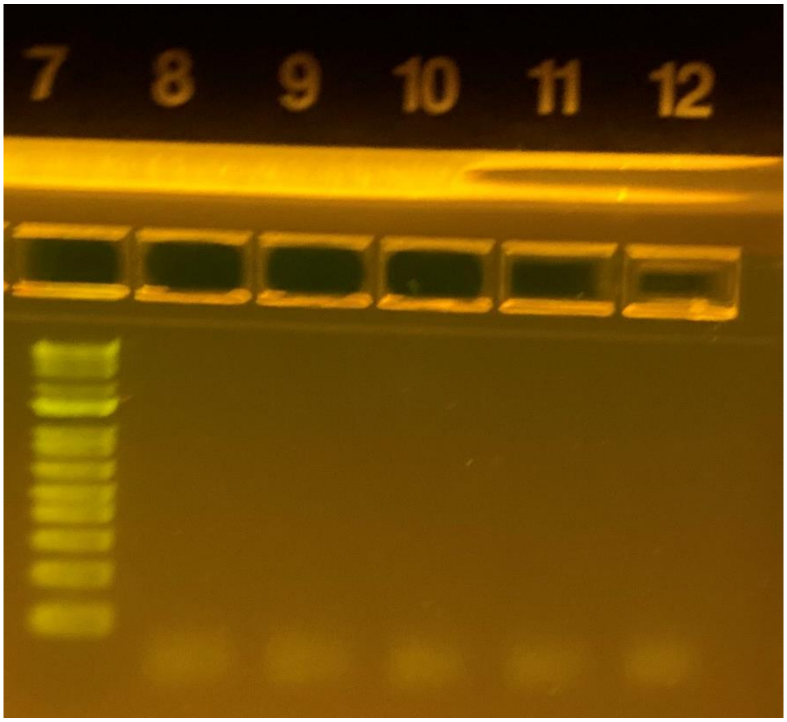

Figure S16. HB196 PCR results on HB503-enriched *D. fuscus* DNA templates, Lanes 11-12 NTC

*Accession Information: Northern Dusky Salamander

RAP3641	GSU-27996	5 November 2024	adult	Virginia	Powhatan

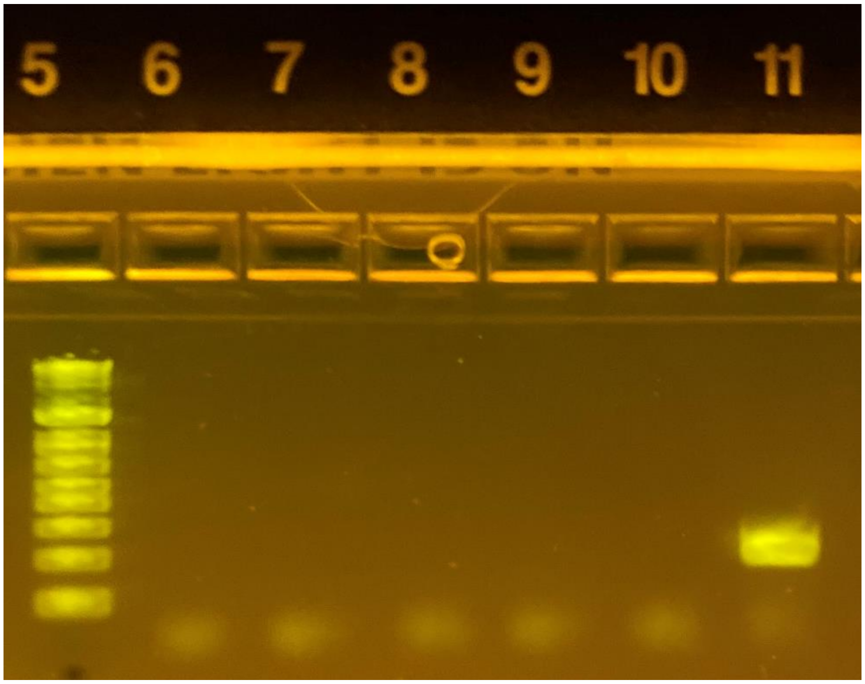

Figure S17. HB196 PCR results directly on *N. maculosus* templates (no HB503 enrichment), Lane 10 NTC, Lane 11 positive control
